# Magnetic mesocellular foams with nickel complexes: as efficient and reusable nanocatalysts for the synthesis of symmetrical and asymmetrical diaryl chalcogenides†

**DOI:** 10.1039/d1na00822f

**Published:** 2022-04-07

**Authors:** Zeinab Shirvandi, Amin Rostami, Arash Ghorbani-Choghamarani

**Affiliations:** Department of Chemistry, Faculty of Science, University of Kurdistan Zip Code 66177-15175 Sanandaj Iran a.rostami@uok.ac.ir; Department of Organic Chemistry, Faculty of Chemistry, Bu-Ali Sina University 65178-38683 Hamedan Iran a.ghorbani@basu.ac.ir arashghch58@yahoo.com

## Abstract

In this work, magnetic mesocellular foam (M-MCF) silica nanoparticles were prepared *via* inserting magnetic nanoparticles into the pores of mesocellular foams, the inner surface of which was functionalized with a methionine–nickel complex (M-MCF@Met–Ni). The structure of the as-prepared nanocatalysts was studied by FT-IR spectroscopy, BET, TGA, VSM, SEM, HR-TEM, EDS, WDX, XRD, and ICP-OES techniques. Thereafter, this nanocatalyst was used as a new, effective, and magnetically reusable catalyst for C–S and C–Se bond formation under mild conditions. All corresponding products were prepared with good yields and appropriate turnover number (TON) and turnover frequency (TOF), which reveals the high activity of this magnetic nanocatalyst in both reactions. In addition, the recovery and hot filtration tests indicated that this catalyst could be simply separated from the reaction mixture using an outside magnet and reused five consecutive times without any significant loss of its catalyst activity or metal leaching.

## Abstract 2

In this work, magnetic mesocellular foam (M-MCF) nanoparticles were prepared *via* inserting magnetic nanoparticles into the pores of mesocellular foams, the inner surface of which was functionalized with a methionine–nickel complex (M-MCF@Met–Ni).

## Graphical abstract

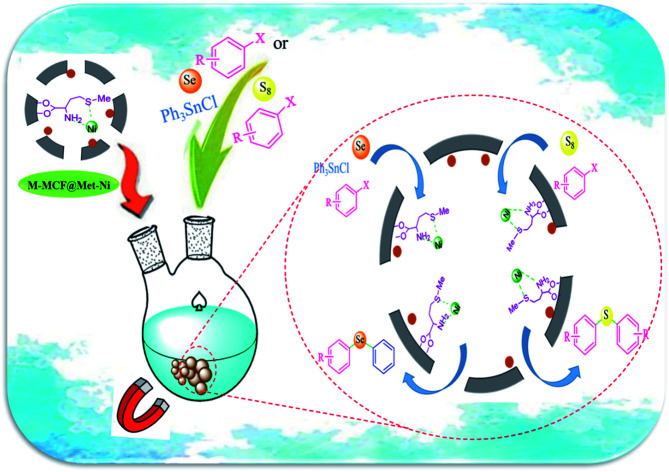

## Introduction

The simple recovery and reusability of the catalyst are essential factors in catalytic processes and industrial applications.^1–4^ In a homogeneous catalytic system, the activity and selectivity of the catalyst are better, but the difficulty in catalyst isolation from the reaction mixture creates limitations.^5–8^ Therefore, the immobilization of homogeneous catalysts on solid materials has attracted the attention of chemists in recent years. However, the low activity of heterogeneous catalysts is their major restriction.^9–13^ Nowadays, to combine the advantages of both homogeneous and heterogeneous catalysts, nanoparticles have been used as excellent and efficient catalyst supports.^14–16^ Among the various nanoparticles, mesoporous, silica compounds especially siliceous mesocellular foams (MCFs) due to a three-dimensional mesoporous structure with internal connectivity, large pore size (diameter 20–50 nm), uniform pore size ranges, large pore volumes, high surface areas (>1000 m^2^ g^−1^), a large number of silanol groups that contribute to high catalyst loading and high thermal and chemical stability are good candidates for catalyst supports.^17–20^ However, the separation of mesoporous silica catalysts from the reaction mixture due to their small size is frequently arduous and costly by filtration methods. This difficulty could be solved by incorporating magnetite nanoparticles into the unique cage-like pores of siliceous MCFs. The resulting magnetic MCF possesses not only the unique features of mesoporous materials such as high surface area and high catalyst loading capacity but also magnetic properties that enable it to be simply separated by an external magnetic field and recycled. Therefore, these features make it a promising support for the immobilization of catalysts.^21–26^

The C–S and C–Se coupling reactions are of high importance in organic synthesis, pharmaceutical industry as well as materials science.^27–29^ Sulfides are used as valuable intermediates in the production of several potent drugs for the treatment of Alzheimer's and Parkinson's diseases, diabetes, and inflammatory and immune diseases.^30–33^ In general, different methods and catalysts for the synthesis of diaryl chalcogenides have been identified, one of the most practical pathways being cross-coupling reactions between aryl halides and thiols/diselenides catalyzed over transition metals.^34–37^ However, these procedures require harsh reaction conditions such as high temperatures and expensive, toxic and polar organic solvents. Furthermore, the use of volatile and foul-smelling thiols causes serious industrial and environmental issues. However, the oxidative homocoupling of thiols results in the formation of unwanted disulfides and can act as catalyst deactivators.^38–40^ To overcome these drawbacks, various sulfur surrogates, such as carbon disulfide,^41,42^ thiourea,^43,44^ sodium thiosulfate,^45,46^ potassium thiocyanate,^47,48^ sulfur powder,^49,50^ and 1,3-oxathiolane^51^ have been used to substitute thiols in the synthesis of sulfides. Toward this aim, selenourea,^52^ potassium selenocyanate,^53,54^ and selenium powder^55,56^ are also used as selenium sources. Among these, the use of sulfur and selenium powders for C–S and C–Se coupling respectively, are more popular due to their low cost, odorless, stability, and availability.

Therefore, introducing new methodologies for the preparation of diaryl sulfides and selenides using sulfur and selenium powders is an urgent requirement. Recently, we have reported odorless and one-pot methods for the synthesis of phenyl aryl sulfides using triphenyltin chloride/S_8_ systems as thiolating agents for the thioetherification of organic (pseudo) halides in the presence of copper salts as homogeneous catalysts.^50,57^ To expand these methods, we became interested in studying the possibility for one-pot selenylation of aryl halides using triphenyltin chloride/Se as a phenylselenating agent in the presence of a heterogeneous catalyst.

In this work, we prepared and characterized a magnetic mesocellular foam-supported methionine–nickel complex (M-MCF@Met–Ni) as a novel magnetically reusable nanocatalyst for the synthesis of symmetrical diaryl sulfides and phenyl aryl selenides. The present methods are superior to other currently available methods due to the use of triphenyltin chloride/Se as a phenylselenating agent and M-MCF@Met–Ni as a heterogeneous green nanocatalyst for the first.

## Experimental

### General information

All substances and solvents were purchased from Aldrich and Merck Chemical Companies and utilized without further purification. The morphology and particle size of the nanostructures were obtained using an FESEM-TESCAN MIRA3 scanning electron microscope (SEM). High-resolution transmission electron microscopic (HR-TEM) images of the nanostructures were acquired using an FEI Quanta TEC9G20 microscope operating at 200 kV. Furthermore, this scanning electron microscope was used for nanoparticle component elemental analysis (EDS and WDX). Using an X'Pert PRO MPD instrument from Panalytical Company, the X-ray powder diffraction patterns of the nanostructures were performed using Cu Kα radiation at 40 kV and 30 mA. A Shimadzu DTG-60 Thermal Analysis system was used to record the thermogravimetric analysis (TGA) curve of prepared samples in the temperature range of 30–900 °C at a heating rate of 10 °C min^−1^ in air. The textural properties of the nanostructures were detected by a N_2_ adsorption test using an Asap 2020 device from Micromeritics Company. Moreover, the content of nickel in the nanocatalyst was measured by inductively coupled plasma-optical emission spectrometry (ICP-OES) using a 730-ES-Varian device. Magnetic measurements of the prepared samples were done using a vibrating sample magnetometer (VSM, MDKFD, Iran) in magnetic fields up to 20 kOe. Fourier-transform infrared (FT-IR) spectra were recorded from KBr pellets using a VRTEX 70 model BRUKER FT-IR spectrophotometer in the range of 400 to 4000 cm^−1^ with an average of 25 scans per sample. ^1^H NMR spectra were used to confirm the structures of the materials (^1^H NMR, 300 MHz, CDCl_3_, and 500 MHz, DMSO).

### Synthesis of magnetic mesocellular foam (M-MCF) silica

The pure mesocellular foam (MCF) silica was synthesized by a previously reported method.^58–60^ Initially, Pluronic P123 (2 g, 0.4 mmol) was added to an aqueous HCl solution (1.6 M, 75 mL) and stirred to obtain a clear solution. Then, ammonium fluoride (23 mg, 0.6 mmol) and 1,3,5-trimethyl benzene (2 g, 17 mmol) were added as organic swelling agents. The solution was stirred at 35–40 °C for 45 min. After that, tetraethoxysilane (4.4 g, 21 mmol) was added and the reaction mixture was again stirred for 20 h, and subsequently transferred into an autoclave and kept at 110 °C for 24 h. The resulting white precipitate was filtered, washed with deionized water, and dried at 60 °C. Finally, the obtained solid was calcined at 550 °C in the air for 5 h. The magnetic particles were inserted into the pores of the foam using modified procedures for the preparation of the magnetic mesocellular foam silica.^61,62^ For this purpose, 1 g of foam was added to 5 mL of a methanol solution containing Fe(NO_3_)_3_·9H_2_O (2.68 g); the mixture was dried in an oven at 80 °C. Then, propionic acid (4.6 mL) was added to the Fe(NO_3_)_3_-impregnated foam, and the mixture was stirred at 90 °C for 3 h to obtain an iron propionate complex. Finally, for the decomposition of the iron propionate complex, the resulting sample was heated to 300 °C in the air (1 °C min^−1^) and kept at this temperature for 30 min. The solid product was denoted as M-MCF.

### Synthesis of methionine–nickel(ii) complex immobilized on M-MCFs

First, the obtained M-MCF (1 g) was dispersed in deionized water (30 mL) by sonication for 30 min, and then methionine (2.5 mmol) was added to this mixture. Next, the reaction mixture was refluxed in a nitrogen atmosphere at 90 °C for 48 h. After that, the obtained solid (M-MCF@Met) was separated using an external magnet, washed several times with distilled water and ethanol, and dried at 50 °C. Finally, the obtained M-MCF@Met (1 g) was sonicated in ethanol (30 mL) and Ni(NO_3_)_2_·6H_2_O (2 mmol) was added to the reaction mixture and refluxed in a nitrogen atmosphere for 24 h. After completion of the reaction, the resulting product was collected using a magnet, washed several times with ethanol, and dried at 50 °C.

### General procedure for the synthesis of diaryl sulfides using aryl halides and S_8_

First, 40 mg of M-MCF@Met–Ni was added to a mixture of aryl halide (1 mmol), S_8_ (1 mmol), and KOH (6 mmol) in DMSO. The mixture was stirred at 120 °C, and the progress of the reaction was checked by TLC. After completion of the reaction, the mixture was cooled and the catalyst was separated using a magnet. The residue was extracted with water and ethyl acetate, and the organic phase was dried over Na_2_SO_4_. After solvent evaporation and purification on a silica gel (*n*-hexane–EtOAc), the products were obtained in 70–97% yields.

### General procedure for the synthesis of phenyl aryl selenides using aryl halides and triphenyltin chloride

Triphenyltin chloride (0.5 mmol), aryl halide (1 mmol), Se (1 mmol), K_2_CO_3_ (4 mmol), and 50 mg of M-MCF@Met–Ni as catalyst were mixed in PEG-200 (2 mL) at 100 °C. The progress of the reaction was checked by TLC; when the reaction was complete, the reaction mixture was cooled to room temperature. Then, the catalyst was collected using an external magnet and the reaction mixture was extracted with diethyl ether. After that, the resulting organic phase was washed with water and dried on anhydrous Na_2_SO_4_. After solvent evaporation and purification by column chromatography on a silica gel (*n*-hexane/EtOAc), the products were obtained in 69–96% yields.

### Selected spectral data

#### Di(*p*-tolyl)sulfide^63^

^1^H NMR (300 MHz, CDCl_3_): *δ* (ppm) = 7.28 (d, *J* = 8.2 Hz, 4H), 7.14 (d, *J* = 8 Hz, 4H), 2.36 (s, 6H).

#### 4,4′-Dimethoxy diphenyl sulfide^64^

^1^H NMR (300 MHz, CDCl_3_): *δ* (ppm) = 7.27 (d, *J* = 6.9 Hz, 4H), 6.87 (d, *J* = 8.7 Hz, 4H), 3.89 (s, 6H).

#### Diphenyl selenide^65^

^1^H NMR (500 MHz, DMSO): *δ* (ppm) = 7.65–7.63 (m, 4H), 7.35–7.30 (m, 6H).

#### Phenyl(*p*-tolyl)selenide^65^

^1^H NMR (300 MHz, CDCl_3_): *δ* (ppm) = 7.66–7.63 (m, 2H), 7.46–7.43 (m, 2H), 7.28–7.22 (m, 3H), 7.09 (d, *J* = 7.9 Hz, 2H), 2.37 (s, 3H).

## Result and discussion

**Scheme 1:**
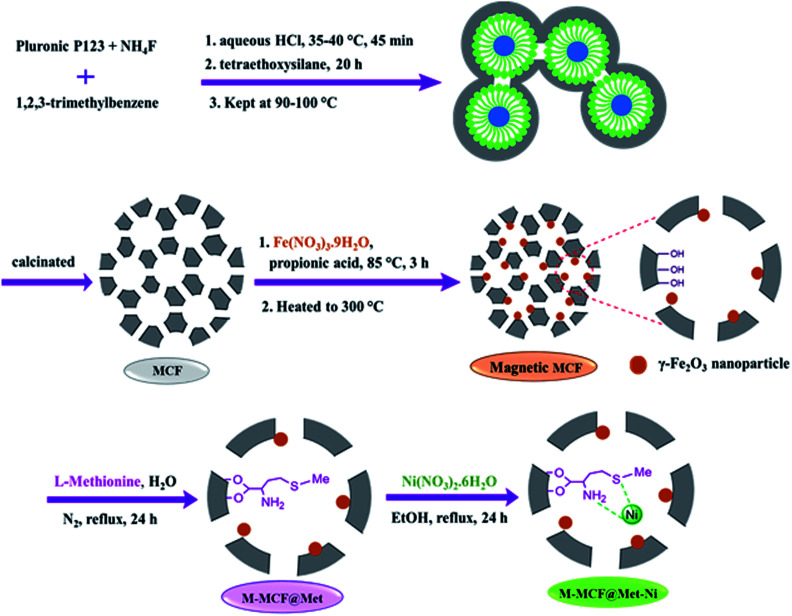
Synthesis of M-MCF@Met–Ni.

The method of immobilization of the nickel complex onto the porous surface of magnetic mesocellular foam (M-MCF) silica nanoparticles for the synthesis of M-MCF@Met–Ni is presented in Scheme 1. Initially, the pure mesocellular foam (MCF) silica was synthesized based on previous reports.^58,59^ The magnetic nanoparticles were then doped into the pores of the MCF.^61,62^ Next, the surface of the M-MCF nanoparticles was functionalized with methionine as the ligand (M-MCF@Met). In the final step, M-MCF@Met–Ni magnetic nanocatalysts were obtained by reacting nickel with M-MCF@Met. The structure of MCF@Met–Ni as a magnetically recoverable nanocatalyst was characterized by TGA, FT-IR spectroscopy, VSM, SEM, HRTEM, WDX, EDS, XRD, BET, and ICP-OES techniques.

### Catalyst characterizations

**Fig. 1:**
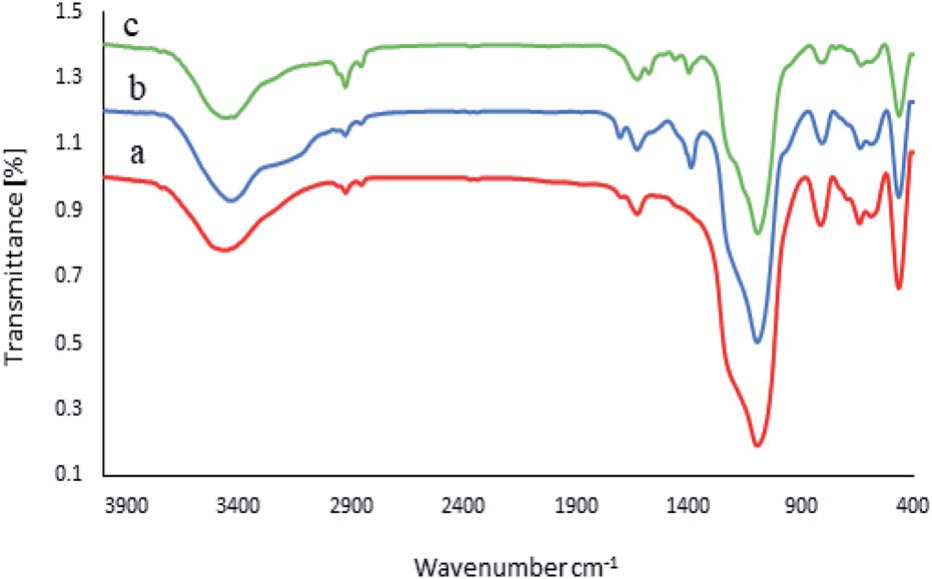
FT-IR spectra of (a) M-MCF, (b) M-MCF@Met and (c) M-MCF@Met–Ni.

The FT-IR spectra of the M-MCF, M-MCF@Met, and M-MCF@Met–Ni are shown in Fig. 1, within the frequency range of 400–4000 cm^−1^. In the FT-IR spectrum of the M-MCF, the absorption peaks at 584 and 642 cm^−1^ are assigned to the Fe–O bond stretching vibration, those at 459, 811, and 1085 cm^−1^ are assigned to the symmetric and asymmetric vibrations of Si–O–Si bonds in silica, and the absorption peak at 3456 cm^−1^ is assigned to the O–H bond stretching vibration (Fig. 1a). In Fig. 1b, the peak at around 2854–2925 cm^−1^ is assigned to the aliphatic C–H stretching vibration. Moreover, the appearance of two peaks at 1380 and 1706 cm^−1^ could be attributed to the C–N and C=O stretching vibrations respectively. The bond of bending of N–H appeared at 1625 cm^−1^. The spectra of M-MCF@Met showed that methionine was successfully modified on the surface of M-MCF magnetic nanoparticles. In Fig. 1c, the N–H bending bond of methionine shifted to lower wavenumbers due to the coordination of the N groups of the ligand with a Ni metal.

**Fig. 2:**
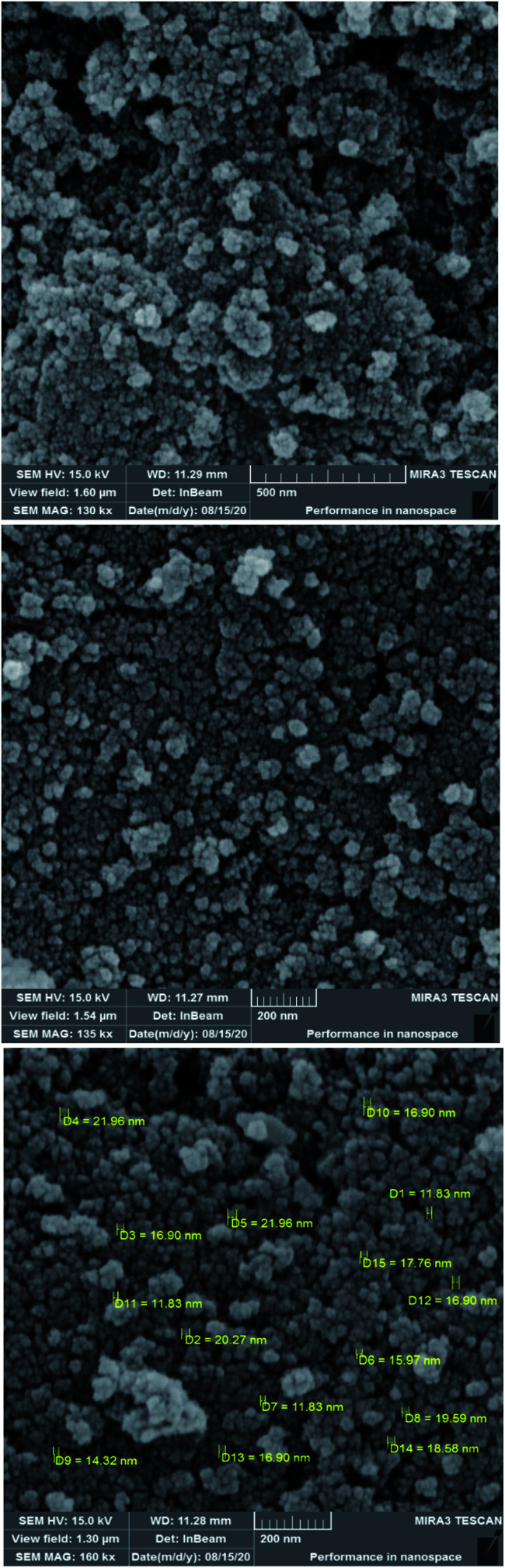
SEM images of M-MCF@Met–Ni.

**Fig. 3:**
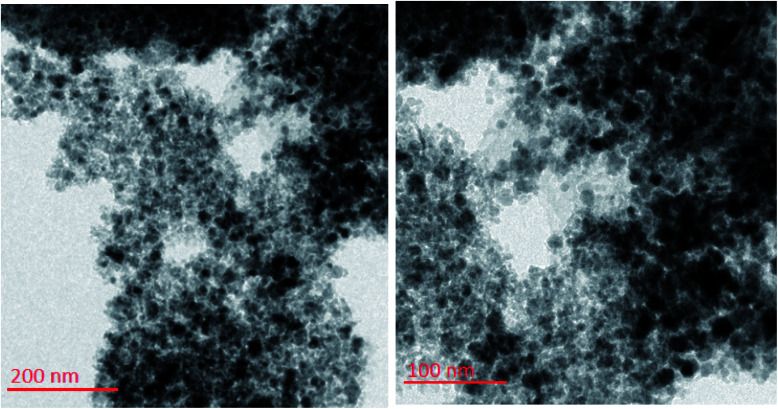
HR-TEM images of M-MCF@Met–Ni.

**Fig. 4:**
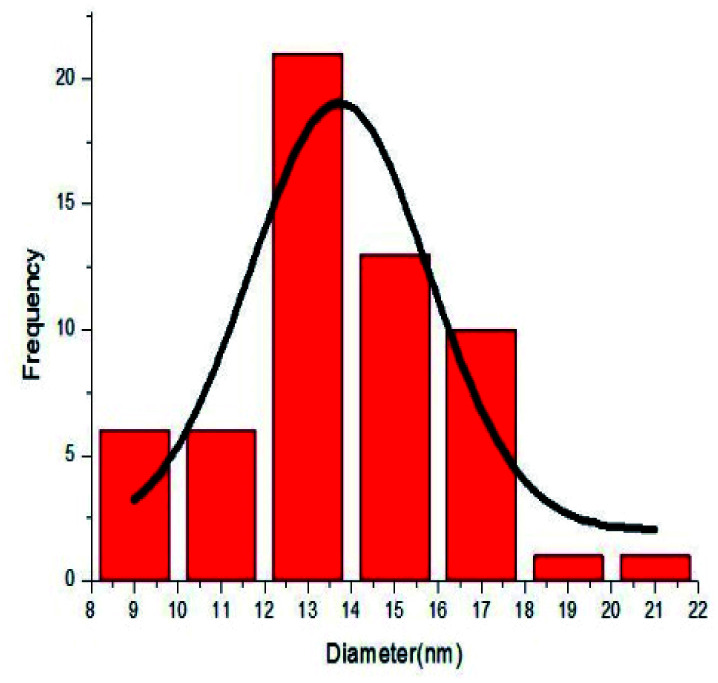
Particle size distribution histogram of M-MCF@Met–Ni.

To investigate the morphology and particle size of the catalyst, the surfaces of the M-MCF@Met–Ni were analysed by scanning electron microscopy (SEM). The SEM images of M-MCF@Met–Ni in Fig. 2 indicate that the majority of the particles have identical quasi-spherical shapes. Moreover, the catalyst was prepared of particles in the size range of 18–30 nm. To get more detailed information about the morphology of M-MCF@Met–Ni as well as the distribution and size of γ-Fe_2_O_3_ particles, the nanocomposite was surveyed by the HR-TEM technique (Fig. 3). As can be seen, the HR-TEM images of M-MCF@Met–Ni nanocomposites reveal a disordered array of MCF silica consisting of large cell pores with a uniform sized distribution. The HR-TEM images also clearly show that γ-Fe_2_O_3_ spherical nanoparticles (dark spots) are located inside the pores of the MCF silica.^66^ Moreover, the γ-Fe_2_O_3_ particle size distribution histogram is shown in Fig. 4. It can be seen that the particle size of γ-Fe_2_O_3_ is between 12 and 16 nm, which agrees with the particle size calculated from XRD data using the Debye–Scherrer equation.

**Fig. 5:**
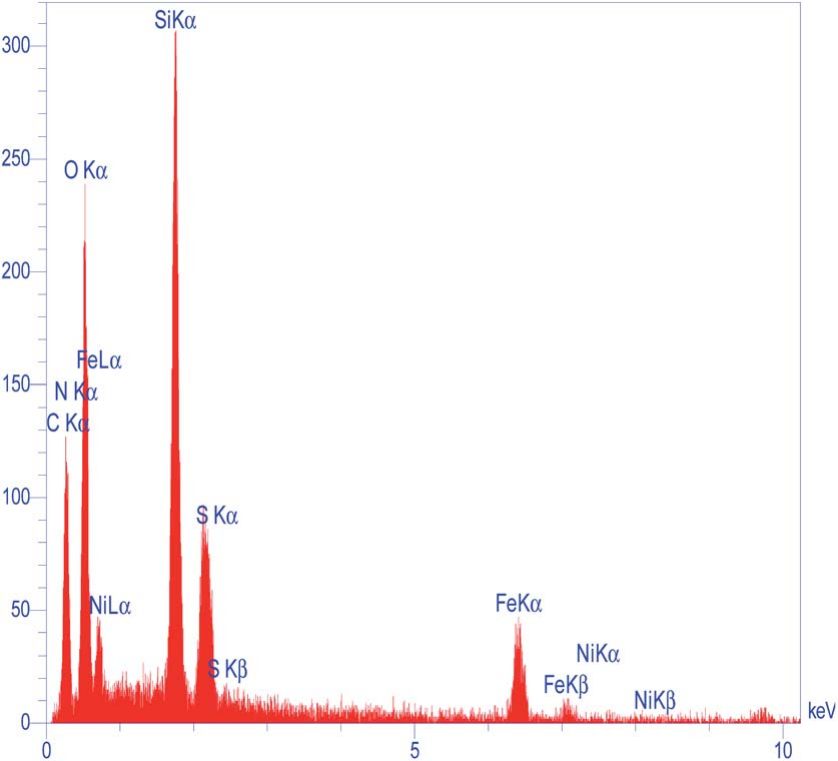
EDS spectrum of M-MCF@Met–Ni.

**Fig. 6:**
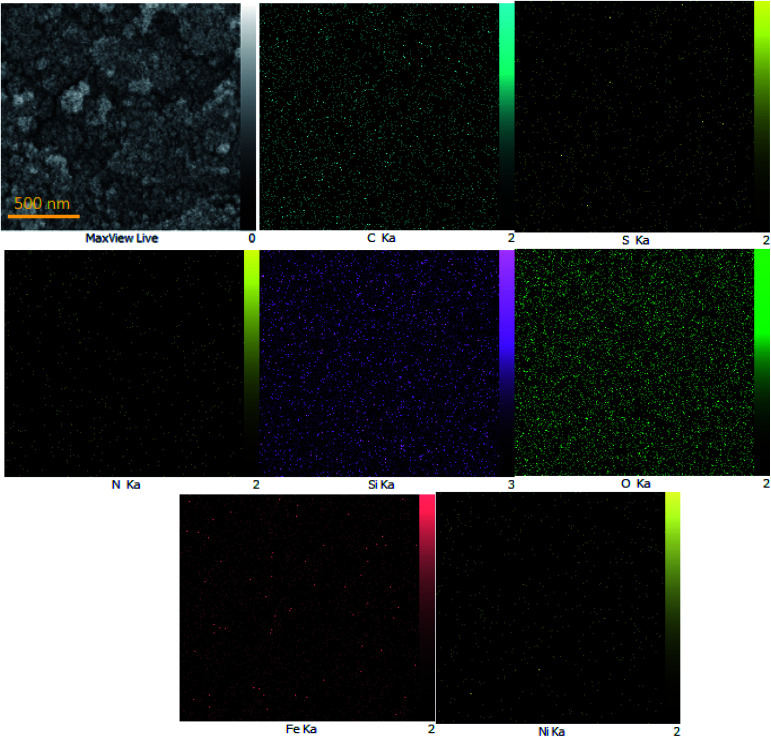
Elemental mapping of M-MCF@Met–Ni.

To determine the presence of various elements in the catalyst, the energy-dispersive X-ray spectroscopy (EDX) analysis of M-MCF@Met–Ni was carried out. As depicted in Fig. 5, the EDX spectrum confirms the existence of N, C, Fe, Si, O, S, and Ni elements in the catalyst structure. In X-ray mapping analysis, the homogeneous distribution of all elements found in the structure of this catalyst is visible, as shown in Fig. 6. Furthermore, the exact amounts of Fe in the catalyst and Ni loaded on the surface of M-MCF@Met using ICP analysis were found to be 4.4 mmol g^−1^ and 0.037 mmol g^−1^, respectively.

**Fig. 7:**
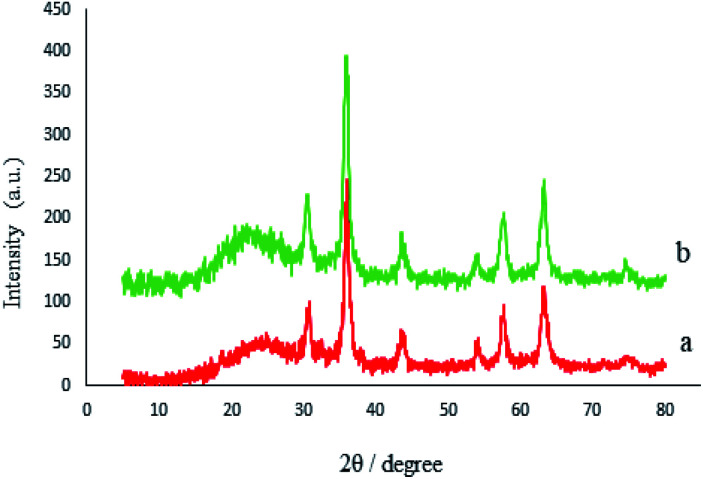
XRD patterns of (a) M-MCF and (b) M-MCF@Met–Ni.

The crystalline structures of the M-MCF particles and M-MCF@Met–Ni were determined by X-ray diffraction (XRD), as shown in Fig. 7. The XRD pattern of the M-MCF indicates six peaks of 2*θ* values at 30.6°, 36.0°, 43.7°, 54.1°, 57.6°, and 63.2°, which correspond to the standard pattern of γ-Fe_2_O_3_ nanoparticles and indicate the presence of magnetic nanoparticles within the foam pore. The broad peak of 2*θ* at 16–28° represents the presence of amorphous silica in the M-MCF structure.^61^ Furthermore, the presence of these peaks in M-MCF@Met–Ni shows that the surface of M-MCF nanoparticles was successfully functionalized with nickel complex and the γ-Fe_2_O_3_ phase was preserved during the surface modification of the M-MCF.

**Fig. 8:**
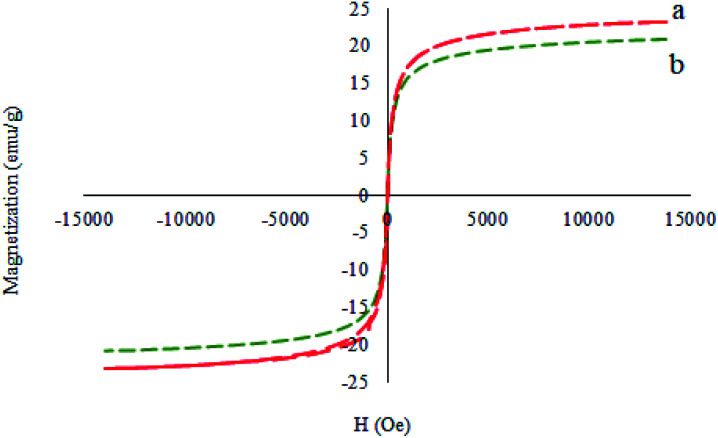
Magnetization curves for (a) M-MCF and (b) M-MCF@Met–Ni.

Fig. 8 illustrates the magnetic properties of the M-MCF and M-MCF@Met–Ni measured using a vibrating sample magnetometer at room temperature (VSM). These curves display that the VSM measurement for M-MCF@Met–Ni (20.91 emu g^−1^) is lower than that of M-MCF nanoparticles (22.24 emu g^−1^), which is due to the existence of organic layers and nickel complexes supported on M-MCF magnetic nanoparticles.

**Fig. 9:**
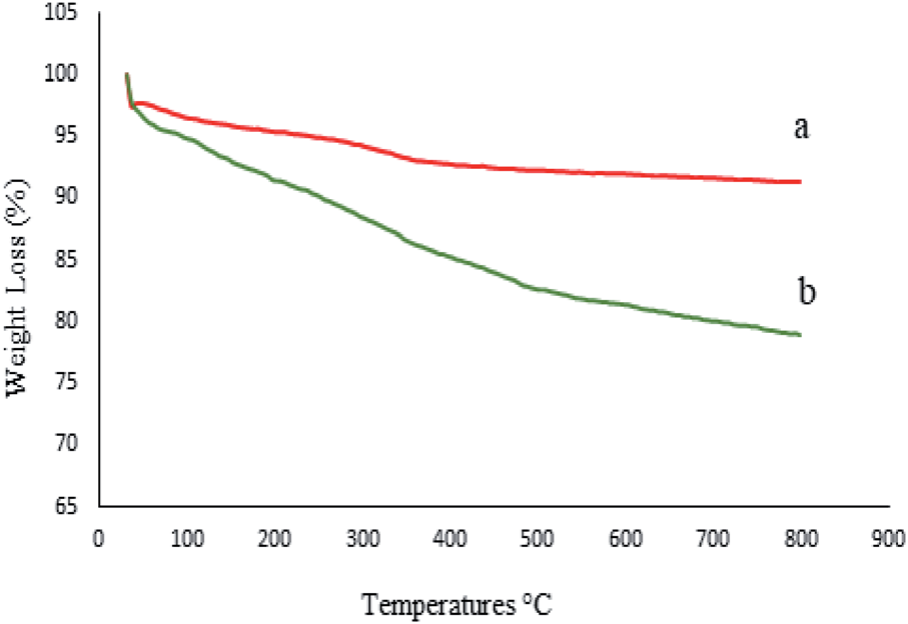
TGA diagrams of (a) M-MCF and (b) M-MCF@Met–Ni.

To investigate the thermal stability of M-MCF@Met–Ni and calculate the amount of organic groups supported on the surface of M-MCF magnetic nanoparticles, thermo-gravimetric analysis (TGA) was used. Fig. 9 shows the TGA curve of the M-MCF and M-MCF@Met–Ni. Based on the TGA curve of the M-MCF, the weight loss of about 2.3% below 200 °C is due to the evaporation of physically adsorbed water. M-MCF also shows a weight loss of approximately 3.5% in the range of 200–500 °C related to the condensation reaction between the surface Si–OH groups.^58^ The TGA curve of M-MCF@Met–Ni shows a weight loss of about 3.4% below 200 °C, corresponding to the removal of the adsorbed water and organic solvent. The second weight loss of about 13% between 200 and 655 °C is attributed to the decomposition of immobilized organic groups on the surface of M-MCF magnetic nanoparticles.

**Fig. 10:**
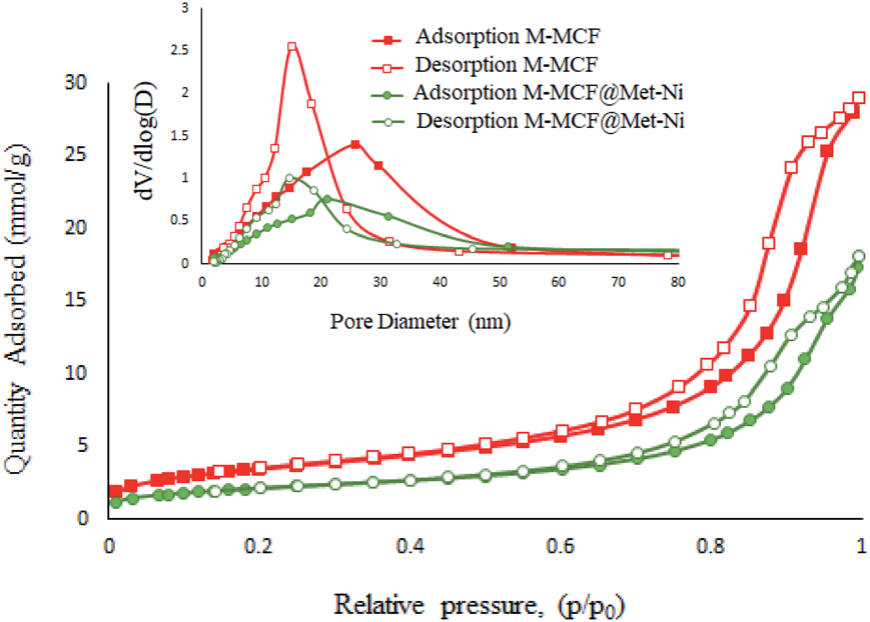
N_2_ adsorption–desorption isotherm of M-MCF and M-MCF@Met–Ni.

Fig. 10 presents the N_2_ adsorption–desorption isotherms of the M-MCF and M-MCF@Met–Ni. These materials exhibit type IV isotherms that are characteristic of mesoporous materials according to the IUPAC classification.^62^ Moreover, the obtained textural properties of the samples by the BET technique are summarized in Table 1. As shown in Table 1, the BET surface area, specific pore volume, window size and cell size for M-MCF are 285.95 m^2^ g^−1^, 1.01 cm^3^ g^−1^, 15.01 nm, and 25.85 nm, respectively. After the functionalization of the M-MCF, these values change to 171.90 m^2^ g^−1^, 0.63 cm^3^ g^−1^, 14.62 nm, and 20.02 nm, respectively. As expected, the amount of these parameters in M-MCF@Met–Ni is lower than the amount of M-MCF nanoparticles, which indicates the grafting of the organic layers and nickel complexes on channels of M-MCF magnetic nanoparticles.

**Table 1:** Texture properties of M-MCF and M-MCF@Met–Ni obtained from N_2_ adsorption–desorption studies

Sample	*S*_BET_ (m^2^ g^−1^)	Pore diameter (nm)		*V*_total_ (cm^3^ g^−1^)
		Window (nm)	Cell (nm)	
M-MCF	285.95	15.01	25.85	1.01
M-MCF@Met–Ni	171.90	14.62	20.02	0.63

### Catalytic studies

The catalytic activity of M-MCF@Met–Ni was investigated for C–S and C–Se bond formation. Initially, the C–S coupling reaction of aryl halides with S_8_ as the sulfur source was investigated in the presence of M-MCF@Met–Ni. To optimize the reaction conditions, the coupling of iodobenzene with S_8_ was considered as the sample reaction. Different parameters such as base, temperature, solvent, and amount of the catalyst were optimized (Table 2). To check the effect of the solvent, the reaction of the model was tested in various solvents such as DMSO, PEG-200, water, DMF, and dioxane, among which DMSO was most effective (Table 2, entry 3). The reaction was tested in the presence of different amounts of M-MCF@Met–Ni. As shown in Table 2, the best yield of the product was observed in the presence of 40 mg of catalyst. By increasing the amount of catalyst from 40 up to 60 mg, no significant change in product yield was observed (Table 2, entry 7). Then, the effect of temperature (Table 2, entries 10 and 11) and nature of the base (Table 2, entries 12–15) on the sample reaction were evaluated and the best results were found using KOH as an effective base at 120 °C. With optimal conditions in hand (Table 2, entry 3), the reactions of different derivatives of aryl halides (aryl iodide, aryl bromide, aryl chloride) with electron-donating and electron-withdrawing functional groups and S_8_ were performed to synthesize symmetrical diaryl sulfides. Turnover number (TON) and turnover frequency (TOF) parameters were used to evaluate the stability and activity of the catalysts. To calculate the TON and TOF, the following formulas were used. Turnover number = number of moles of products per mole of catalyst precursor; TOF = TON per hour. As shown in Table 3, the designed products were obtained in good to excellent yields with good TON and TOF, showing the high catalytic activity of M-MCF@Met–Ni for these reactions. Moreover, the experimental results show that aryl iodides and bromides react more quickly than aryl chlorides.

**Table 2:** Optimization of the reaction conditions for the coupling reaction of iodobenzene with S_8_ in the presence of M-MCF@Met–Ni

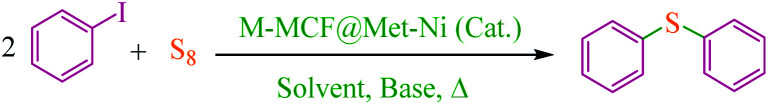						
Entry	Catalyst (mg)	Solvent	Base (6 mmol)	Temp. (°C)	Time (min)	Yield^a^ (%)
1	—	DMSO	KOH	120	90	N.R
2	40	PEG	KOH	120	90	N.R
3	40	DMSO	KOH	120	90	97
4	40	H_2_O	KOH	Reflux	90	N.R
5	40	Dioxane	KOH	Reflux	90	N.R
6	40	DMF	KOH	120	90	20
7	60	DMSO	KOH	120	90	97
8	20	DMSO	KOH	120	90	73
9	10	DMSO	KOH	120	90	56
10	40	DMSO	KOH	100	90	87
11	40	DMSO	KOH	80	90	74
12	40	DMSO	NaOH	120	90	76
13	40	DMSO	Et_3_N	120	90	N.R
14	40	DMSO	name	120	90	N.R
15	40	DMSO	Na_2_CO_3_	120	90	N.R

a Isolated yield.

**Table 3:** Catalytic C–S coupling reaction of aryl halides with S_8_ in the presence of M-MCF@Met–Ni

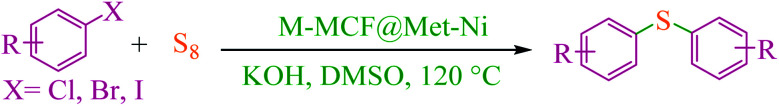							
Entry	Ar–X	Product	Time (h)	Yield^a^ (%)	TON	TOF	Mp (°C)^ref.^
1	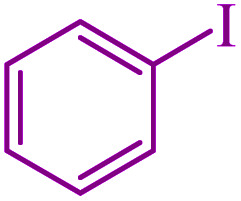	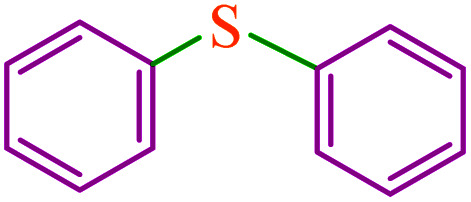	1.5	97	655.4	436.9	Oil^67^
2	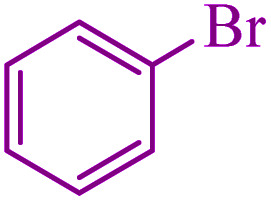	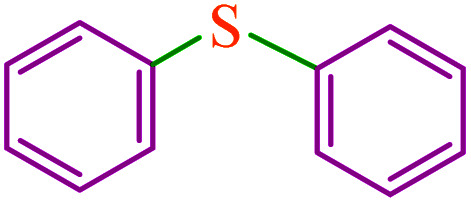	4	85	574.3	143.5	Oil^67^
3	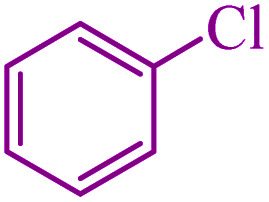	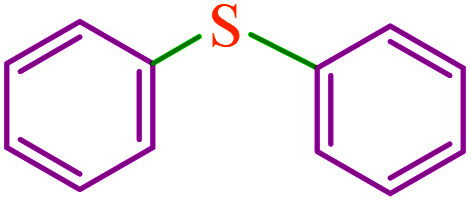	12	80	540.5	45.0	Oil^67^
4	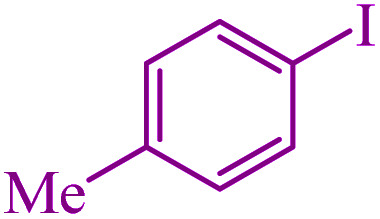	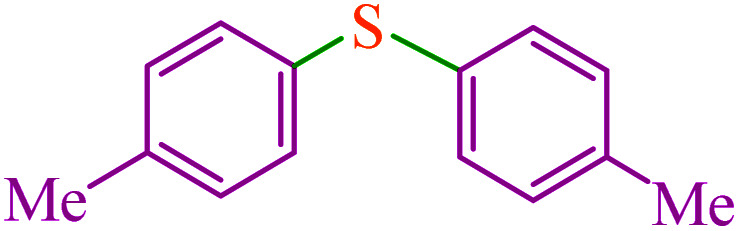	2.15	83	560.8	260.8	53–54 (ref. 35)
5	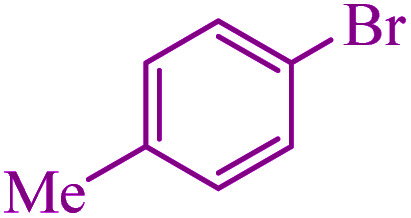	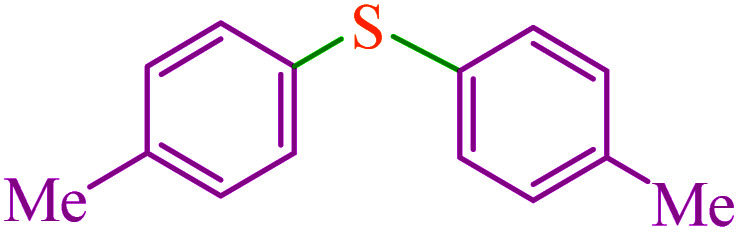	4.5	79	533.7	118.6	53–54 (ref. 35)
6	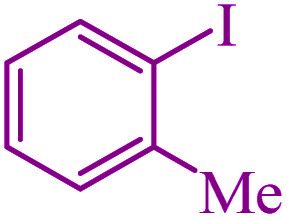	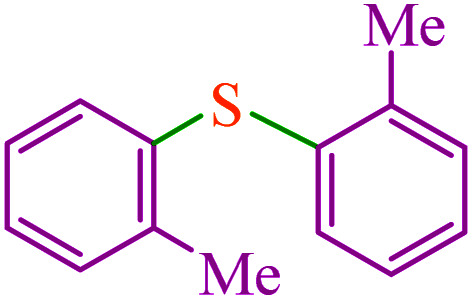	6	81	547.2	91.2	56–57 (ref. 68)
7	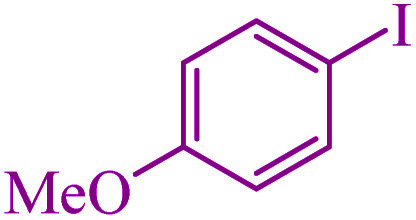	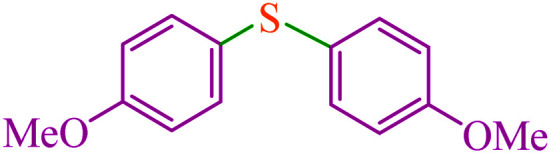	3.15	93	628.3	199.4	Oil^69^
8	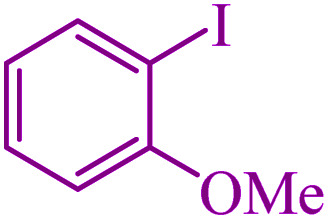	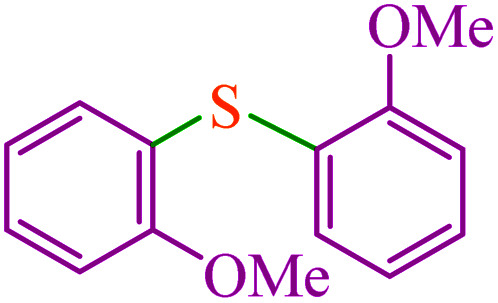	4.5	89	601.3	133.6	Oil^64^
9	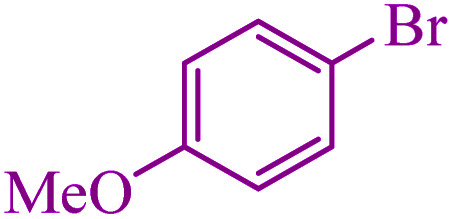	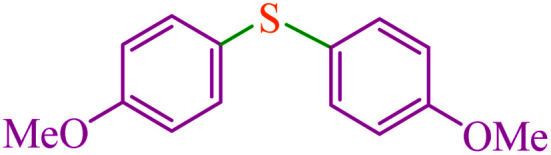	5.5	90	608.1	110.5	Oil^69^
10	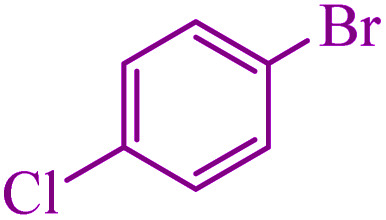	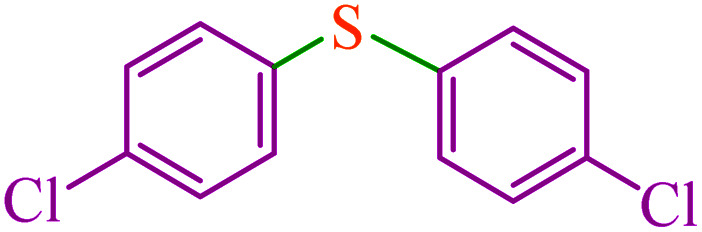	6	86	581.0	96.8	Oil^35^
11	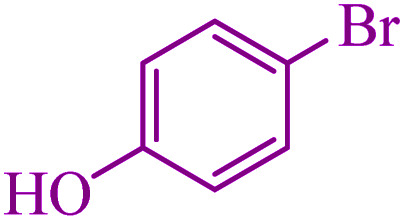	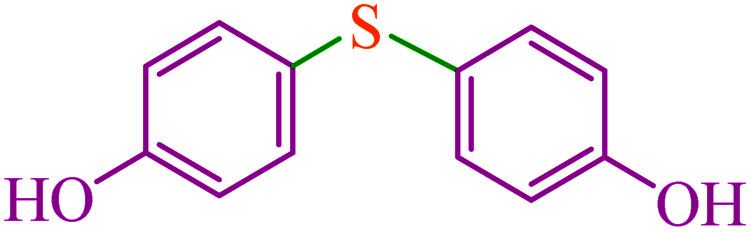	6.4	84	567.5	85.2	152–154 (ref. 64)
12	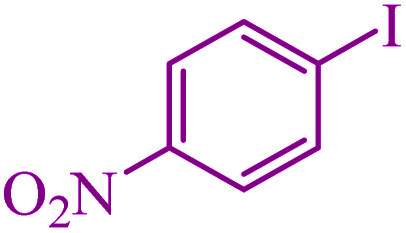	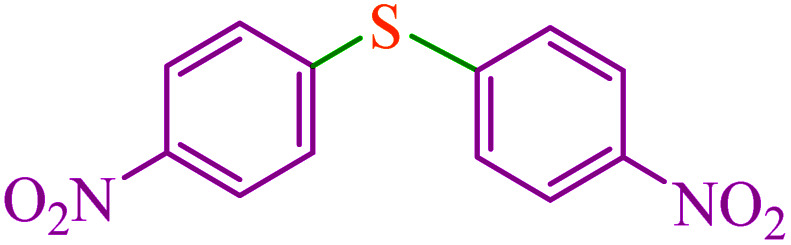	3	90	608.1	202.7	155–157 (ref. 50)
13	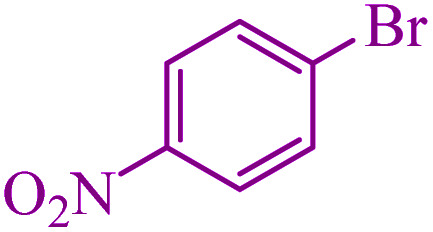	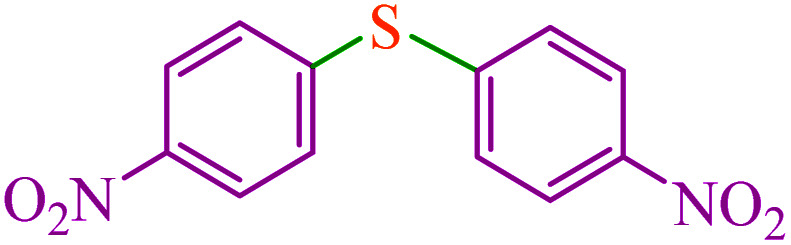	4.5	83	560.8	124.6	155–157 (ref. 50)
14	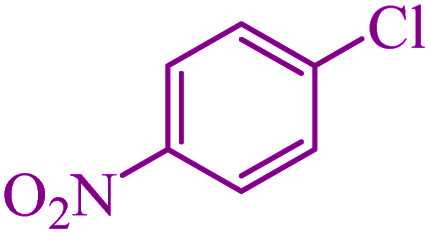	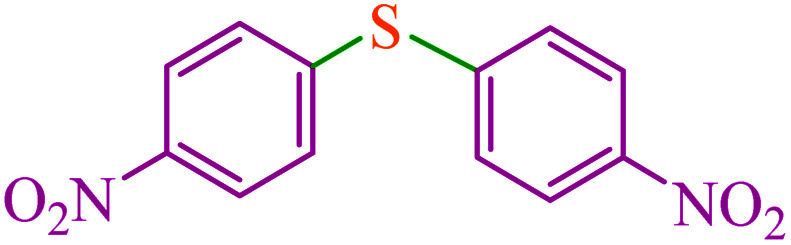	12	70	472.9	39.4	155–157 (ref. 50)
15	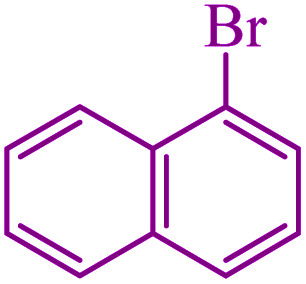	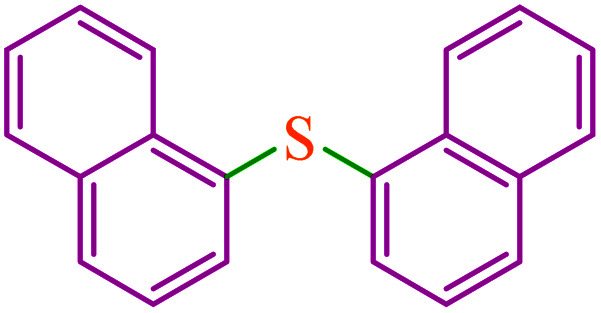	5.5	80	540.5	98.2	110–112 (ref. 42)

a Isolated yield.

**Scheme 2:**
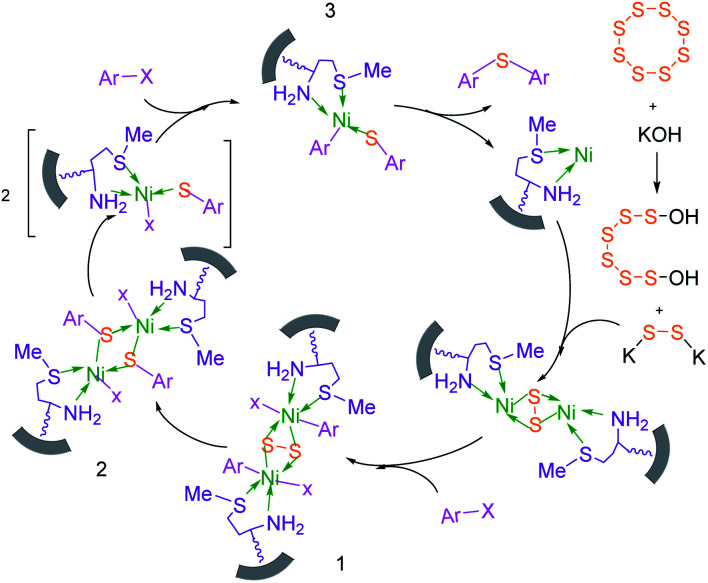
Suggested mechanism for the C–S coupling reaction in the presence of M-MCF@Met–Ni.

Based on our previously reported results,^67,68^ the plausible mechanism for the synthesis of sulfides in the presence of M-MCF@Met–Ni as the catalyst is presented in Scheme 2. In the first stage, S_8_ reacts with KOH to form K_2_S_2_. K_2_S_2_ then reacts with M-MCF@Met–Ni to produce the nickel disulfide. In the next step, aryl halide is added to nickel disulfide *via* an oxidative addition reaction to generate intermediate 1, which is converted into intermediate 2 by the migration of the aryl group. After that, the oxidative addition of an aryl halide with intermediate 2 forms intermediate 3, which upon reductive elimination gives the diaryl sulfide product, and the nickel catalyst is also regenerated.

Recently, we have developed efficient methods for C–S bond formation using triphenyltin chloride as a source of the phenyl group.^49^ These investigations and obtained results for the C–S coupling reaction in the presence of M-MCF@Met–Ni prompted us to investigate the catalytic activity of M-MCF@Met–Ni for the synthesis of phenyl aryl selenides *via* a C–Se bond formation reaction of aryl halides with triphenyltin chloride as a phenyl group source using a Se powder as the selenium source. To determine the optimal reaction conditions, the coupling of iodobenzene with triphenyltin chloride and Se powder in the presence of M-MCF@Met–Ni as a nanocatalyst and K_2_CO_3_ as a base in PEG as a solvent was selected as the model reaction. The effect of different parameters (such as solvents, catalyst values, bases, and temperatures) was evaluated; the results are summarized in Table 4. First, different amounts of M-MCF@Met–Ni catalysts (10, 30, 50, and 70 mg) were tested, and the best result was obtained with 50 mg of M-MCF@Met–Ni catalyst (Table 4, entries 2–5). Then, the effect of different solvents such as DMF, DMSO, H_2_O, and dioxan was checked on a model reaction. A minor amount of the desired products was achieved in DMSO and DMF (Table 4, entries 6 and 7), whereas PEG-200 showed the best result for this reaction (Table 4, entry 2). Among the different investigated bases (such as KOH, NaOH, Na_2_CO_3,_ and Et_3_N), K_2_CO_3_ was found to be better than other bases (Table 4, entries 10–12). Finally, to evaluate the effect of temperature, the reaction was performed at different temperatures of 70, 100, and 120 °C. According to the presented results, 120 °C was selected as the appropriate temperature for this reaction (Table 4, entries 13 and 14).

**Table 4:** Optimization of the reaction conditions for the reaction of iodobenzene with triphenyltin chloride and Se in the presence of M-MCF@Met–Ni

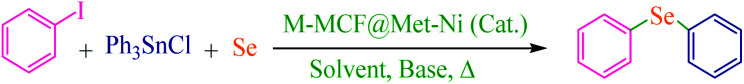						
Entry	Catalyst (mg)	Solvent	Base (4 mmol)	Temp. (°C)	Time (min)	Yield^a^ (%)
1	—	PEG	K_2_CO_3_	100	150	N.R
2	70	PEG	K_2_CO_3_	100	150	96
3	50	PEG	K_2_CO_3_	100	150	96
4	30	PEG	K_2_CO_3_	100	150	70
5	10	PEG	K_2_CO_3_	100	150	52
6	50	DMF	K_2_CO_3_	100	150	76
7	50	DMSO	K_2_CO_3_	100	150	48
8	50	H_2_O	K_2_CO_3_	Reflux	150	N.R
9	50	Dioxan	K_2_CO_3_	Reflux	150	N.R
10	50	PEG	NaOH	100	150	67
11	50	PEG	Et_3_N	100	150	N.R
12	50	PEG	Na_2_CO_3_	100	150	78
13	50	PEG	K_2_CO_3_	120	150	98
14	50	PEG	K_2_CO_3_	80	150	84

a Isolated yield.

**Scheme 3:**
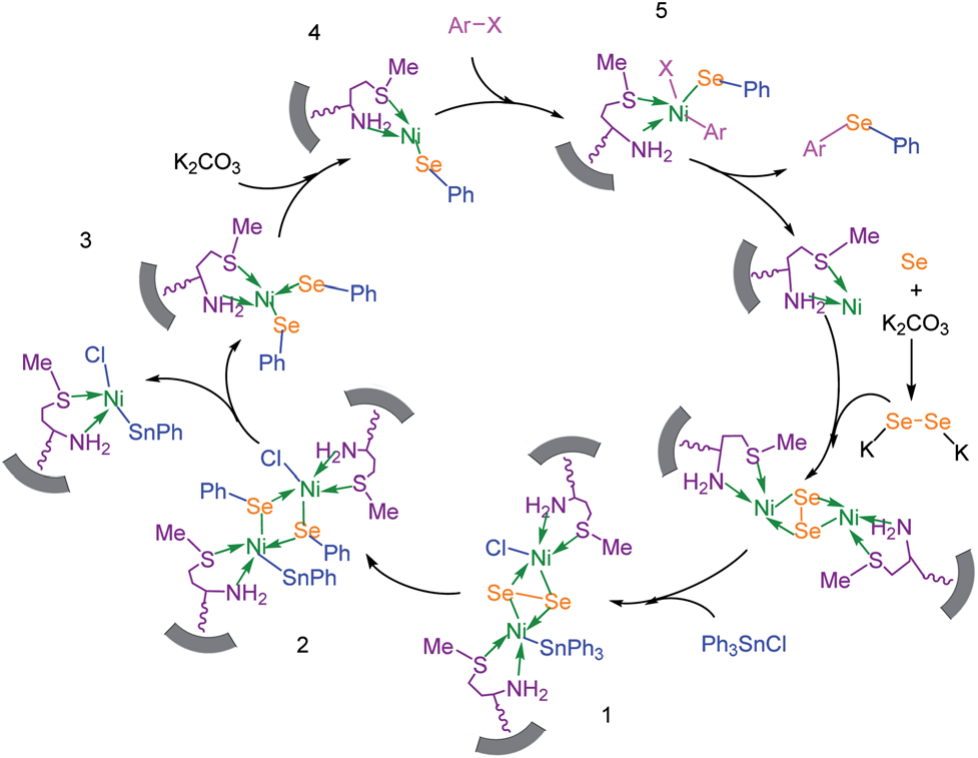
Suggested mechanism for the C–Se coupling reaction in the presence of M-MCF@Met–Ni.

To extend the scope of this process, various derivatives of aryl halides (aryl iodide, aryl bromide, aryl chloride) were tested with triphenyltin chloride and Se in the presence of the catalyst, under optimal reaction conditions. The results of these studies are shown in Table 5; all the corresponding asymmetrical selenides were obtained in good to excellent yields in the range of 69 to 96% and the appropriate TOF. As can be seen, aryl halide derivatives with electron-withdrawing groups show more activity than aryl halides with electron-donating groups. To investigate the chemoselectivity of this method, the reaction of 1-bromo-4-chlorobenzene (as a dihalogenated aryl halide) was performed with triphenyltin chloride and Se; bromide showed a higher reaction (Table 5, entry 10). Although the exact mechanism for the C–Se bond formation *via* reaction of aryl halides with triphenyltin chloride and Se in the presence of MCF@Met–Ni nanocatalyst is not clear at this time, based on the previously reported mechanisms for C–S bond formation using aryl halide, triphenylene chloride and sulfur powder^68–70^ and C–Se coupling reaction using aryl halides and Se powder^71,72^ the proposed mechanism for the synthesis of phenyl aryl selenides in the presence of M-MCF@Met–Ni nanocatalyst is presented in Scheme 3. First, K_2_CO_3_ reacts with elemental Se to produce potassium diselenide, which reacts with M-MCF@Met–Ni to form nickel diselenide.^73^ Nickel diselenide reacts with triphenyltin chloride *via* an oxidative addition reaction to form intermediate 1, which may be converted into intermediate 2 by phenyl group migration. Intermediate 2 is converted into intermediate 3*via* reductive elimination, which is then transformed into intermediate 4 in the presence of K_2_CO_3_.^63^ Then, intermediate 4 reacts with aryl halide *via* oxidative addition to produce intermediate 5. The desired product may be obtained by reductive elimination of intermediate 5. However, the investigation about details of the mechanism is undertaken in our laboratory.

**Table 5:** Catalytic C–Se coupling reaction of aryl halides with triphenyltin chloride and Se in the presence of M-MCF@Met–Ni

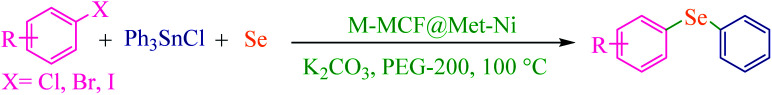							
Entry	Ar–X	Product	Time (h)	Yield^a^ (%)	TON	TOF	Mp (°C)^ref.^
1	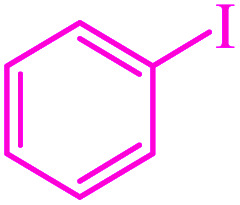	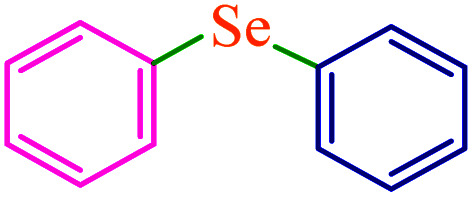	2.5	96	518.9	207.5	Oil^72^
2	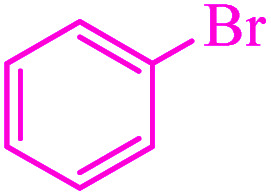	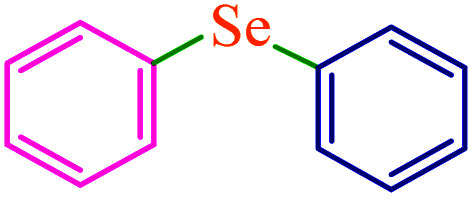	3.5	91	491.8	140.5	Oil^72^
3	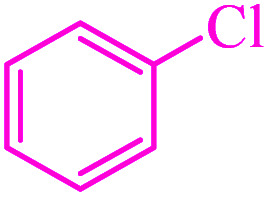	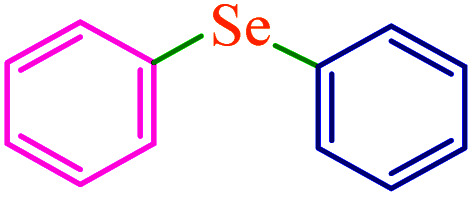	24	86	464.8	19.3	Oil^72^
4	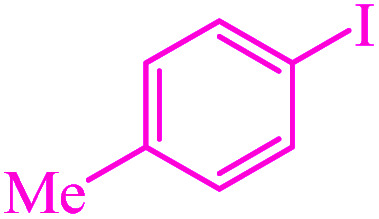	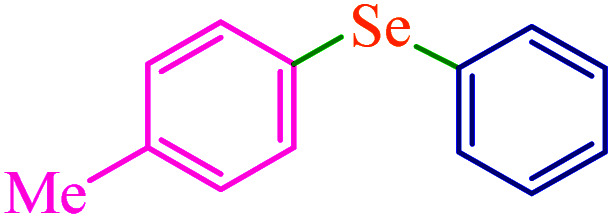	4.5	92	497.2	110.5	Oil^74^
5	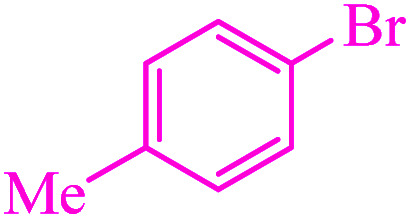	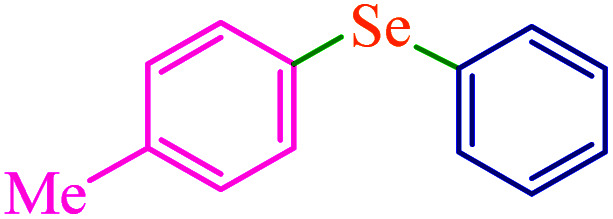	6	90	486.4	81.0	Oil^74^
6	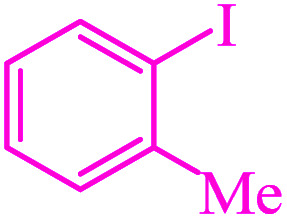	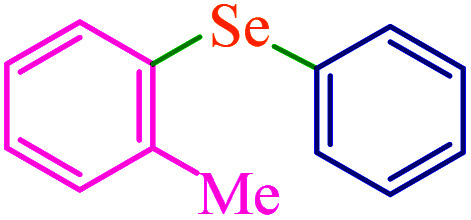	5	85	459.4	91.8	Oil^75^
7	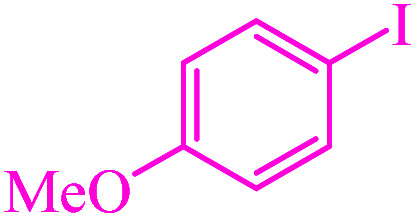	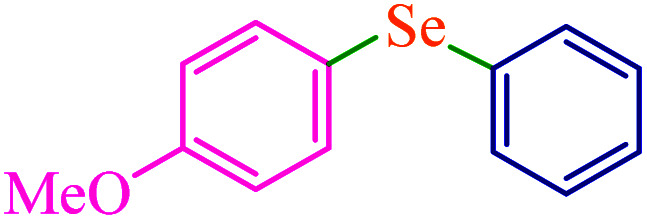	3.75	90	486.4	129.7	Oil^75^
8	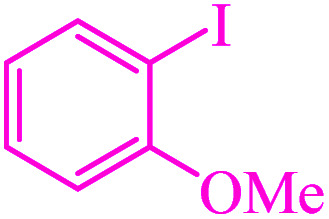	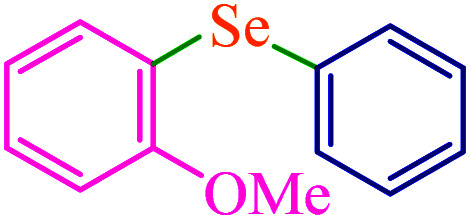	4	83	448.6	112.1	Oil^76^
9	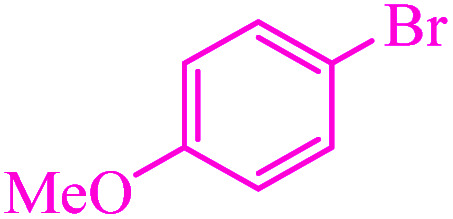	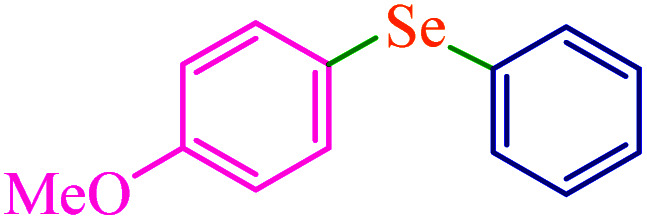	5	86	464.8	92.9	Oil^75^
10	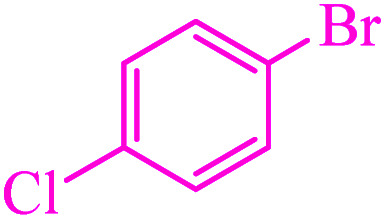	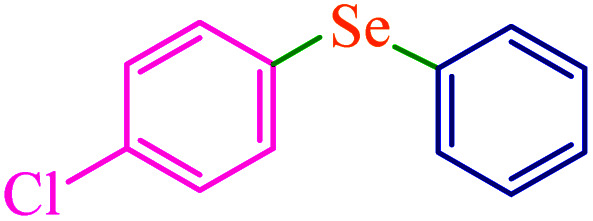	3	90	486.4	162.1	Oil^27^
11	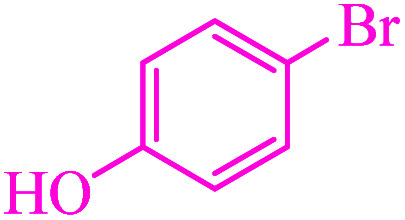	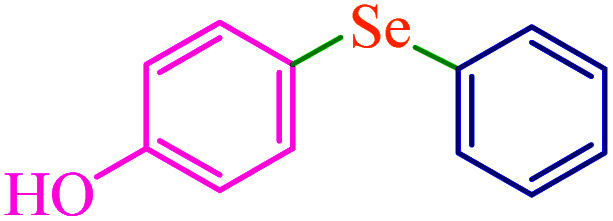	5.5	78	421.6	76.6	Semisolid^27^
12	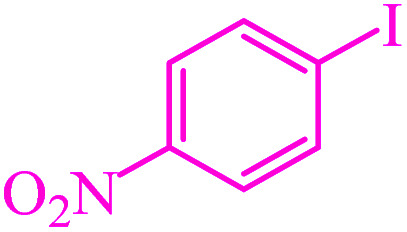	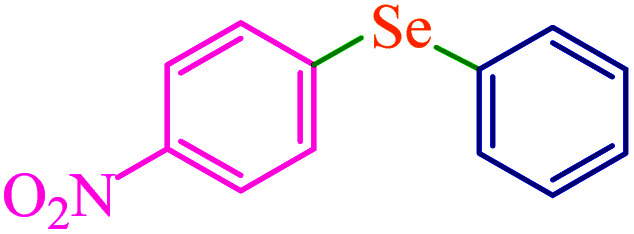	4	90	486.4	121.6	55–57 (ref. 76)
13	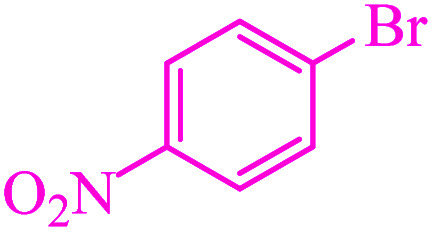	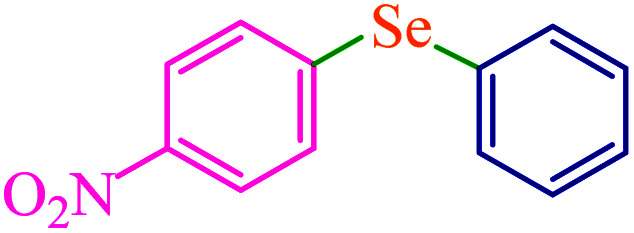	6	88	475.6	79.2	55–57 (ref. 76)
14	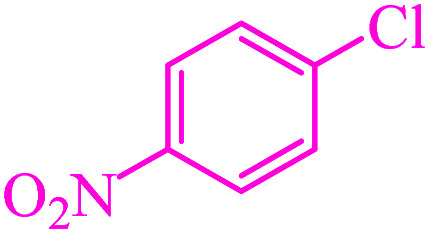	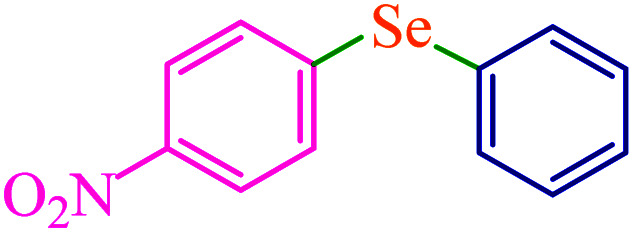	24	69	372.9	15.5	55–57 (ref. 76)
15	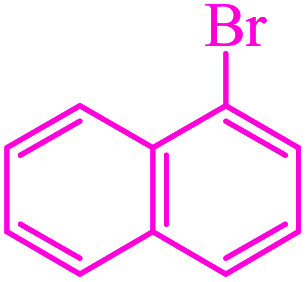	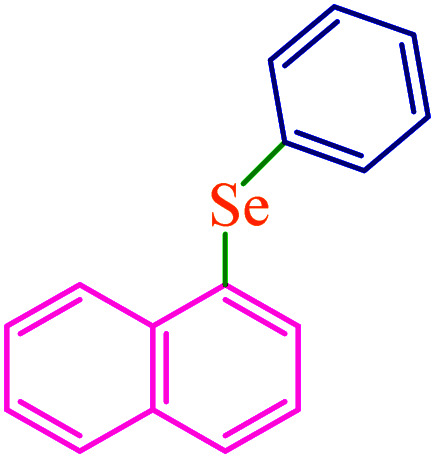	5	85	459.4	91.8	Oil^27^

a Isolated yield.

### Reusability of the catalyst

**Fig. 11:**
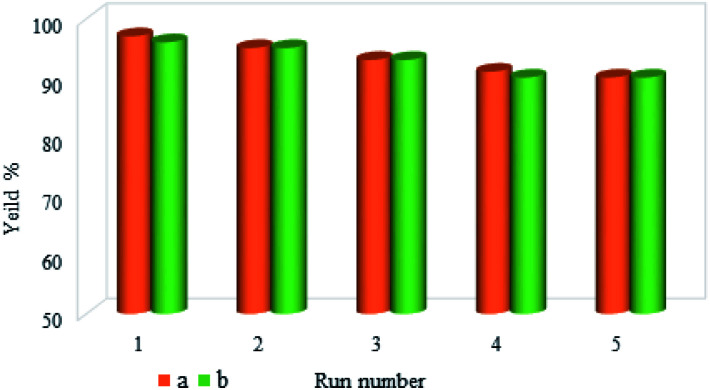
Reusability of the M-MCF@Met–Ni nanocatalyst in both (a) C–S coupling reaction and (b) C–Se coupling reaction.

One of the most important advantages of magnetic catalysts is their reusability, which plays an important role in environmental concerns, and economic and commercial applications. Therefore, the reusability of M-MCF@Met–Ni was studied in the reaction between iodobenzene and S_8_ (for the C–S coupling reaction) and the reaction of iodobenzene with Ph_3_SnCl and Se (for the C–Se coupling reaction). For this purpose, at the end of the reaction, M-MCF@Met–Ni was isolated from the reaction mixture using an external magnet and washed with ethyl acetate and water. The nanocatalyst was then dried and reused under the same reaction conditions. As shown in Fig. 11, M-MCF@Met–Ni was recycled and reused for at least five consecutive runs in both C–S and C–Se coupling reactions without any significant decrease in its catalytic activity.

**Fig. 12:**
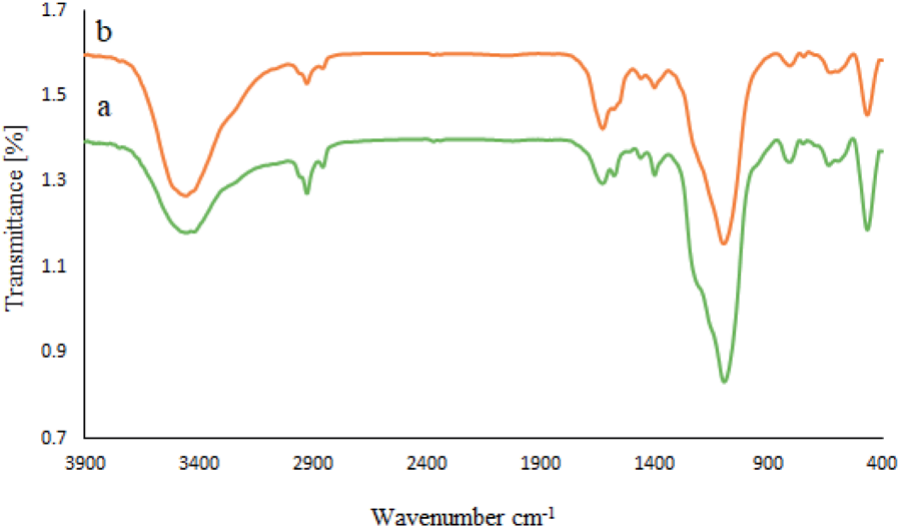
FT-IR spectra of (a) M-MCF@Met–Ni and (b) recovered M-MCF@Met–Ni.

Moreover, the stability of the recycled M-MCF@Met–Ni nanocatalyst after the fifth reuse was investigated by FT-IR spectroscopy, XRD, ICP, SEM, EDX, WDX, TGA, and VSM techniques and compared with a fresh catalyst. The FT-IR spectra of fresh nanocatalyst and recycled nanocatalyst (Fig. 12) show that all peaks of the catalyst structure have remained unchanged even after five runs. Therefore, these results show good stability for M-MCF@Met–Ni after recycling and confirm that this catalyst can be recycled and reused several times without significant changes in its structure.

**Fig. 13:**
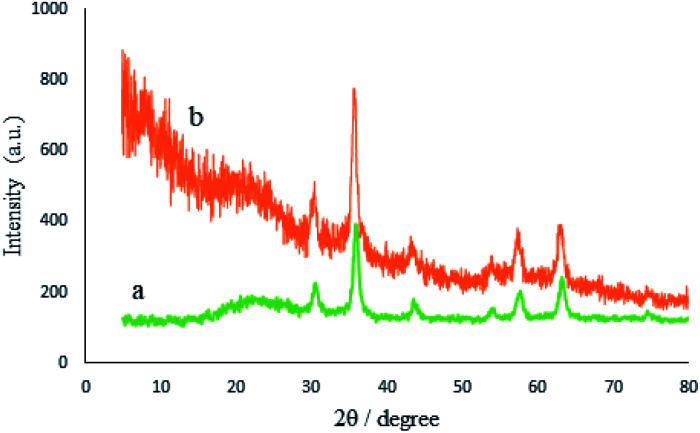
XRD patterns of (a) M-MCF@Met–Ni and (b) recovered M-MCF@Met–Ni.

The crystalline structure of the recycled M-MCF@Met–Ni nanocatalyst is shown in Fig. 13. As it can be seen, the XRD pattern of the recovered catalyst after five runs is the same as that of the fresh catalyst, and the peaks of γ-Fe_2_O_3_ are well preserved. Therefore, the crystalline structure of M-MCF@Met–Ni is stable and has not been changed after five runs of recycling.

In addition, the nickel leaching amount in every cycle was investigated for the synthesis of diphenyl sulfide. By ICP analysis, the amount of nickel on the nanocatalyst surface before and after catalyst recovery was determined, and the results are presented in Table 6. The ICP analysis results indicate that the lowest amount of nickel leaching (0.0008 to 0.001 mmol g^−1^) occurred during the reaction.

**Table 6:** Nickel amounts (mmol g^−1^) analysis of fresh and recovered M-MCF@Met–Ni

Run	1	2	3	4	5
Nickel amounts (mmol g^−1^)	0.03748	0.03668	0.03588	0.03478	0.03348

**Fig. 14:**
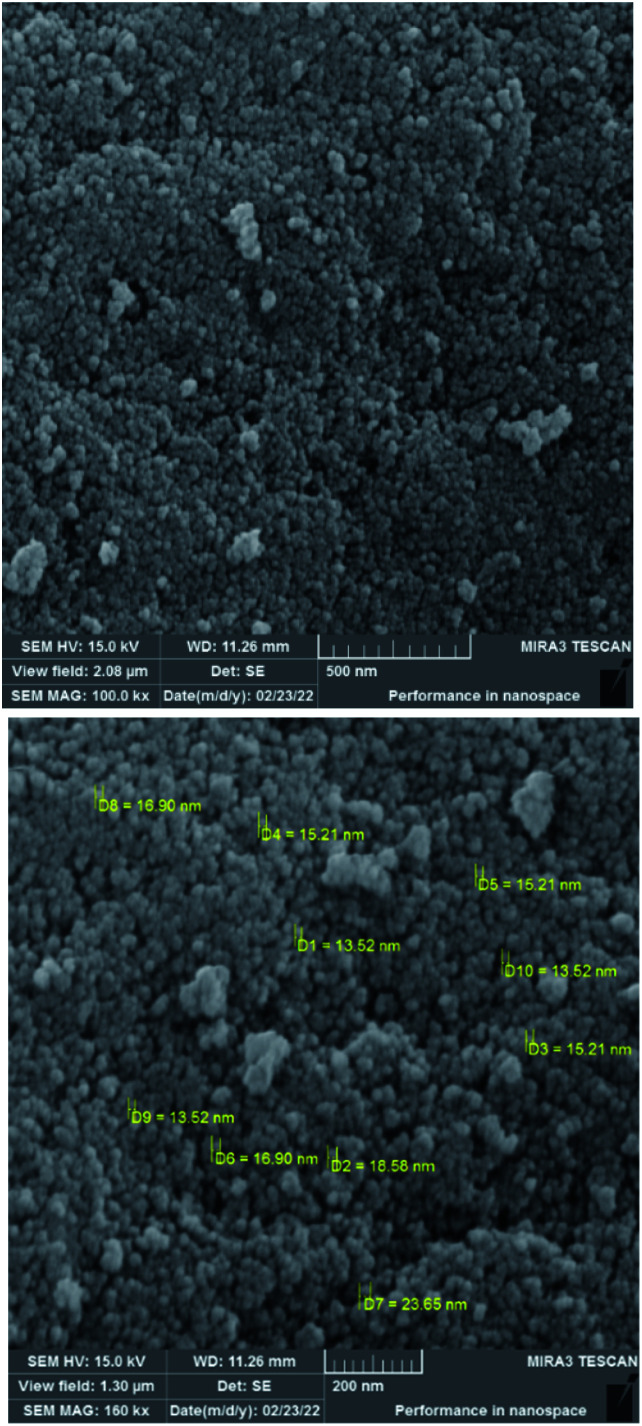
SEM images of recovered M-MCF@Met–Ni.

**Fig. 15:**
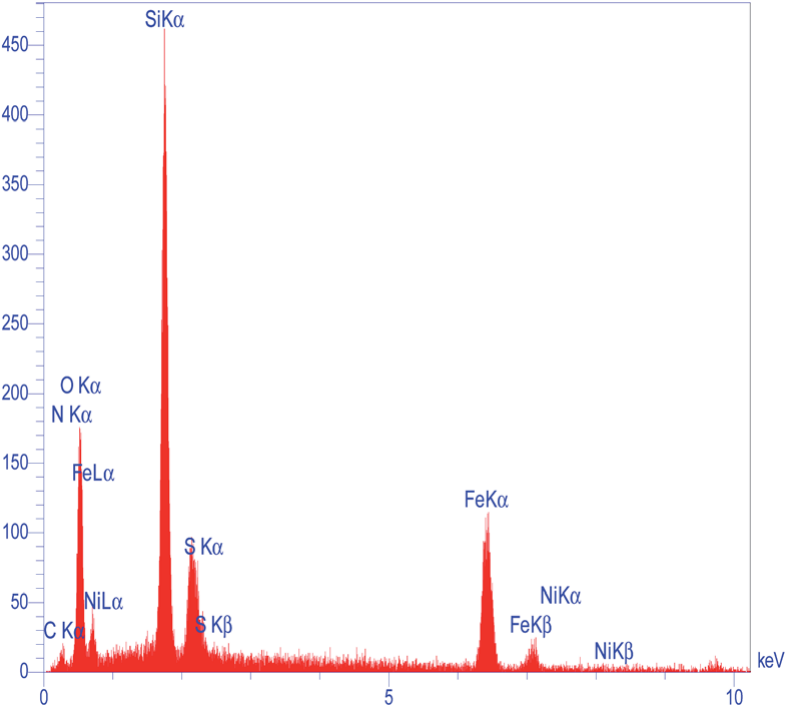
EDS spectrum of recovered M-MCF@Met–Ni.

The morphology and particle size of recovered M-MCF@Met–Ni were investigated by the SEM technique (Fig. 14). The particles of recycled M-MCF@Met–Ni were observed with sizes between 20 and 35 nm, which is similar to the SEM image of the fresh catalyst. These results indicate that the structure of the catalyst was retained after recycling. As shown in Fig. 15, using EDX analysis, the presence of desired elements, including carbon, iron, oxygen, silicon, nitrogen, sulfur, and nickel, in recycled M-MCF@Met–Ni can be observed.

**Fig. 16:**
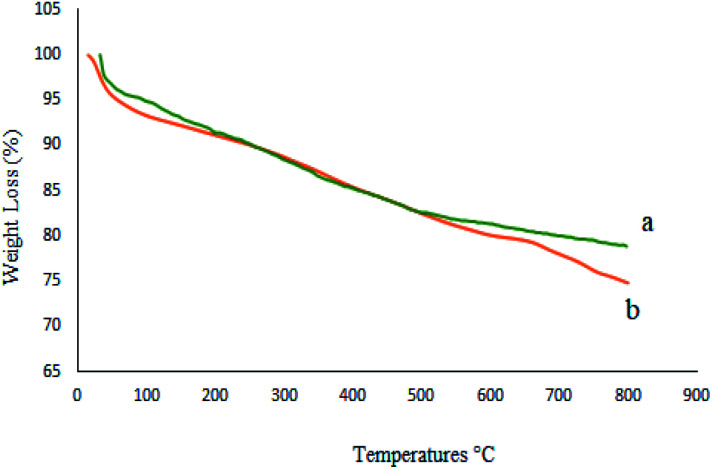
TGA diagrams of (a) M-MCF@Met–Ni and (b) recovered M-MCF@Met–Ni.

The thermal stability of the catalyst was evaluated by TGA of recycled M-MCF@Met–Ni after five reuses (Fig. 16). It can be seen that the TGA data of the catalyst recovered are in good agreement with the fresh catalyst, indicating the thermal stability of the nanocomposite.

**Fig. 17:**
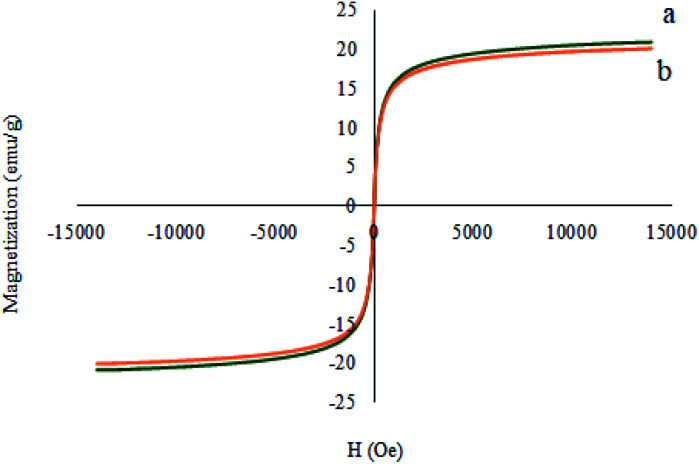
Magnetization curves for (a) M-MCF@Met–Ni and (b) recovered M-MCF@Met–Ni.

The magnetic properties of the recovered catalyst were evaluated using a vibrating sample magnetometer (VSM). In Fig. 17, the magnetization curve of the recycled M-MCF@Met–Ni was measured and compared to the fresh catalyst. As shown in the figure, the magnetization curve of the recycled catalyst after the fifth reuse is similar to the fresh catalyst. These results indicate that the magnetic property of the nanocatalyst was retained during the reaction.

### Leaching test of the catalyst

To investigate the stability and heterogeneity of the synthesized nanocatalyst in the reaction mixture, a hot filtration experiment was performed for coupling of C–Se. In this experiment, the reaction of iodobenzene with triphenyltin chloride and Se powder (Table 5, entry 1) was investigated under optimal conditions, with a reaction duration of 5 h. In half of the reaction time (2.5 hours), the product yield was 57%. The reaction was then repeated and the nanocatalyst was removed after half the reaction time, and the filtrated solution was allowed to react without a catalyst for the remaining time.

The yield of the product was 60% at this stage. These results indicate that significant leaching of nickel has not occurred.

### Comparison of the catalyst

The catalytic activity of M-MCF@Met–Ni was compared with other reported catalysts in the literature for C–S and C–Se reactions. The results of the synthesis of diphenyl sulfide (entry 1–10) and 4-methoxyphenyl-phenyl selenide (entry 11–17) in the presence of M-MCF@Met–Ni, as well as the previously reported methods, are summarized in Table 7. It can be seen that diphenyl sulfide and 4-methoxyphenyl-phenyl selenide were obtained in high yields with good TOF in the presence of M-MCF@Met–Ni. Additionally, M-MCF@Met–Ni has some advantages such as high activity, low cost, non-toxicity, stability, and easy separation using an external magnet.

**Table 7:** Comparison of M-MCF@Met–Ni with previously reported catalysts in the synthesis of diphenyl sulfide and 4-methoxyphenyl-phenyl selenide

Entry	Catalyst	Reaction condition	Time (h)	Yield^a^ (%)	Ref.
1	CuI	Iodobenzene, S_8_, NaOH, PEG-200, 40–60 °C	4.3	93	57
2	Nano-CuFe_2_O_4_	Iodobenzene, thiourea, K_2_CO_3_, DMF, 120 °C	12	94	34
3	CuI–bpy	Iodobenzene, S_8_, Al, MgCl_2_, DMF, 110 °C	22	75	35
4	Cu(ii)-his@CS	Iodobenzene, KSCN, K_2_CO_3_, DMSO, 130 °C	24	90	48
5	NiCl_2_·6H_2_O, 2,2′-bipyridine	Thiophenols, *t*-BuOK, MeCN/DMF, air, r.t.	12	82	52
6	[Cu(MeCN)_4_BF_4_], 2,2′-bipyridine	Sulfonyl hydrazides, 1,2-dichloroethane, air, 120 °C	15	70	52
7	CuI, 1,8-diazabicyclo[5.4.0]undec-7-ene	Iodobenzene, carbon disulfide, toluene, 100 °C	12	85	69
8	PdNP–PNF	Iodobenzene, mercaptobenzothiazole, KOH, DMSO, 130 °C	5	92	77
9	IMes–Cu–Cl	Iodobenzene, thiophenols, LiO^*t*^Bu, toluene, 120 °C	6	81	78
10	M-MCF@Met–Ni	Iodobenzene, S_8_, NaOH, DMSO, 120 °C	1.5	97	This work
11	K_2_S_2_O_8_	Diphenyl diselenide, anisole, THF, r.t.	3	94	27
12	I_2_, MW irradiation	Diphenyl diselenide, anisole, DMSO, 110 °C	0.16	88	30
13	AgNO_3_	Diphenyl diselenide, 4-methoxy-phenyl boronic acid, 1,4-dioxane, air, 100 °C	6	91	37
14	CuI	Diphenyl diselenide, anisole, Cs_2_CO_3_, MeCN, 82 °C	28	95	72
15	CuSO_4_, 1,10-phen.·H_2_O	Diphenyl diselenide, 4-methoxy-phenyl boronic acid, Na_2_CO_3_, EtOH, air, r.t.	5	85	74
16	I_2_, MW irradiation	Diphenyl diselenide, 4-methoxy-phenyl boronic acid, DMSO, 110 °C	0.16	79	75
17	M-MCF@Met–Ni	4-Methoxy-iodobenzene, phenylboronic acid, Se, K_2_CO_3_, PEG-200, 100 °C	3.75	90	This work

a Isolated yield.

## Conclusion

In this study, a novel nanocomposite and magnetically recoverable catalyst (M-MCF@Met–Ni) has been synthesized and its structure has been confirmed by TGA, FT-IR spectroscopy, BET, SEM, EDS, WDX, XRD, VSM, and ICP-OES techniques. Then, the catalytic activity of M-MCF@Met–Ni was examined for the C–S and C–Se bond formation. All products were obtained in significant yields with appropriate TOF values. Moreover, M-MCF@Met–Ni could be recovered easily using an external magnet and recycled for five consecutive times in the mentioned organic reactions. In addition, the significant advantages of the designed system include simple and inexpensive procedure, eco-friendliness, high stability, good to excellent yields of products under relatively mild conditions, and short reaction periods.

## Conflicts of interest

There are no conflicts to declare.

## Supplementary Material

NA-004-D1NA00822F-s001
