# Supplementary material for: Magnetic mesocellular foams with nickel complexes: as efficient and reusable nanocatalysts for the synthesis of symmetrical and asymmetrical diaryl chalcogenides
Source: Nanoscale Adv. 2022 Apr 7;4(9):2208–23. doi: 10.1039/d1na00822f (PMC9419205; doi:10.1039/d1na00822f)
Supplement: NA-004-D1NA00822F-s001 [file NA-004-D1NA00822F-s001.pdf]

Electronic Supplementary Material (ESI) for Nanoscale Advances.
This journal is © The Royal Society of Chemistry 2022

Supporting Information

Functionalized magnetic mescellular foams with nickel complex as a new, green, and reusable nanocatalyst for the synthesis of symmetrical and unsymmetrical diaryl chalcogenide

Zeinab Shirvandi[a], Amin Rostami*[a] and Arash Ghorbani-Choghamarani*[b]

[a.] *Department of Chemistry, Faculty of Science, University of Kurdistan, Zip Code 66177-15175, Sanandaj, Iran. Email: a.rostami@uok.ac.ir*
[b.] *Department of Organic Chemistry, Bu-Ali Sina University, 6517838683 Hamedan, Iran. Email: a.ghorbani@basu.ac.ir; arashghch58@yahoo.com*

Abstruct: Magnetic mescellular foams (M-MCF) silica nanoparticles were prepared by inserting magnetic nanoparticles into the pores of mesocellular foam, then its inner surface was functionalized with methionine-nickel complex (M-MCF@Met-Ni). The structure of the prepared nanocatalyst was studied by FT-IR, BET, TGA, VSM, SEM, EDS, WDX, XRD and ICP-OES techniques. Thereafter, this nanocatalyst was used as a new, effective and magnetically reusable catalyst for the C–S and C–Se bonds formation under mild conditions. All corresponding products were prepared with good yields and appropriate TON and TOF numbers, which reveals the high activity of this magnetic nanocatalyst in both reactions. In addition, the recovery and hot filtration tests indicated that this catalyst could be simply separated from the reaction mixture by an outside magnet and reused five consecutive times without significant loss of its catalyst activity or metal leaching.

Experimental

Synthesis of magnetic mescellular foams silica (M-MCF)

The pure mescellular foams silica (MCF) was synthesized by a previously reported method.[1, 2] Initially, Pluronic P123 (2 g, 0.4 mmol) was added to an aqueous HCl solution (1.6 M, 75 mL) and stirred to obtain a clear solution. Then, ammonium fluoride (23 mg, 0.6 mmol) and 1,3,5 trimethyl benzene (2 g, 17 mmol) as the organic swelling agent were added. The solution was stirred at 35–40 °C for 45 min. After that, tetraethoxysilane (4.4 g, 21 mmol) was added and the reaction mixture was again stirred for 20 h, and subsequently transferred into an autoclave and kept at 110 °C for 24 h. The resulting white precipitate was filtered, washed with deionized water, and dried at 60 °C. Finally, the obtained solid was calcined at 550 °C in air for 5 h. The magnetic particles were inserted into the pores of the foam using modified procedures for the preparation of the magnetic mescellular foams silica.[3, 4] For this purpose, 1 g of foam was added to 5 mL of a methanol solution containing $Fe(NO_3)_3.9H_2O$ (2.68 g); the mixture was dried in an oven at 80 °C. Then, propionic acid (4.6 mL) was added to the $Fe(NO_3)_3$ -impregnated foam, and the mixture was stirred at 90 °C for 3 h to obtain the iron propionate complex. Finally, for the decomposition of the iron propionate complex, the resulting sample was heated to 300 °C in air (1 °C /min) and allowed kept at this temperature for 30 min. The solid product was denoted as M-MCF.

Synthesis of methionine-nickel (II) complex immobilized on M-MCF

First, the obtained M-MCF (1 g) was dispersed in deionized water (30 mL) by sonication for 30 min, then methionine (2.5 mmol) was added to this mixture. Next, the reaction mixture was refluxed under a nitrogen atmosphere at 90 °C for 48 h. After that, the obtained solid (M-MCF@Met) was separated by an external magnet, washed several times with distilled water and ethanol, and dried at 50 ºC. Finally, the obtained M-MCF@Met (1 g) was sonicated in ethanol (30 mL) and the $Ni(NO_3)_2$ (2 mmol) was added

to the reaction mixture, and refluxed under nitrogen atmosphere for 24 h. After completion of the reaction, the resulting product was collected with a magnet, washed with ethanol several times and dried at 50 °C.

General procedure for the synthesis of diaryl sulfides using aryl halides and S_8

40 mg of M-MCF@Met-Ni was added to a mixture of aryl halide (1 mmol), S_8 (1 mmol) and KOH (6 mmol) in DMSO. The mixture was stirred at 120 °C, and the progress of the reaction was checked by TLC. After completion of the reaction, the mixture was cooled and the catalyst was separated using a magnet. The residue was extracted with water and ethyl acetate, and the organic phase dried over Na_2SO_4. After solvent evaporation and purification on a silica gel (n-hexane–EtOAc), the products were obtained in 70-97% yields.

General procedure for the synthesis of phenyl aryl selenides using aryl halids and triphenyltin chloride

Triphenyltin chloride (0.5 mmol), aryl halide (1 mmol), Se (1 mmol), K_2CO_3 (4 mmol) and 50 mg of M-MCF@Met-Ni as catalyst were mixed in PEG-200 (2 mL) at 100 °C. The progress of the reaction was checked by TLC; when the reaction was complete, the reaction mixture was cooled to room temperature. Then the catalyst was collected by an external magnet and the reaction mixture was extracted with diethyl ether. After that, the resultant organic phase was washed with water and dried on anhydrous Na_2SO_4. After solvent evaporation and

Selected spectral data

Di(p-tolyl) sulfide[5]. ^{1}H NMR (300 MHz, $CDCl_3$): δ (ppm)= 7.28 (d, *J*= 8.2 Hz, 4H), 7.14 (d, *J*= 8 Hz, 4H), 2.36(s, 6H).

4,4'-Dimethoxy diphenyl sulfide[6]. ^{1}H NMR (300 MHz, $CDCl_3$): δ (ppm)= 7.27(d, *J*= 6.9 Hz, 4H), 6.87 (d, *J*= 8.7 Hz, 4H), 3.89 (s, 6H).

Diphenyl selenide[7]. ^{1}H NMR (500 MHz, DMSO): δ (ppm)= 7.65-7.63 (m, 4H), 7.35–7.30 (m, 6H).

Phenyl(p-tolyl) selenide[7]. ^{1}H NMR (300 MHz, $CDCl_3$): δ (ppm)= 7.66-7.63 (m, 2H), 7.46-7.43 (m, 2H), 7.28-7.22 (m, 3H), 7.09 (d, *J*= 7.9 Hz, 2H), 2.37 (s, 3H).

Notes and references

1. S. Moradi, Z. Shokri, N. Ghorashi, A. Navaee and A. Rostami, *J. Catal.*, 2020, **382**, 305-319.
2. L. Wei, Y. Zhao, Y. Zhang, C. Liu, J. Hong, H. Xiong and J. Li, *J. Catal.*, 2016, **340**, 205-218.
3. Z. Shokri, N. Azimi, S. Moradi and A. Rostami, *Appl. Organomet. Chem.*, 2020, **34**, e5899.
4. D. Lee, J. Lee, H. Lee, S. Jin, T. Hyeon and B. M. Kim, *Adv. Synth. Catal.*, 2006, **348**, 41-46.
5. A. Rostami, A. Rostami and A. Ghaderi, *J. Org. Chem.*, 2015, **80**, 8694-8704.
6. A. Ghorbani-Choghamarani and Z. Taherinia, *New J. Chem.*, 2017, **41**, 9414-9423.
7. B. Goldani, V. G. Ricordi, N. Seus, E. J. Lenardao, R. F. Schumacher and D. Alves, *J. Org. Chem.*, 2016, **81**, 11472-11476.

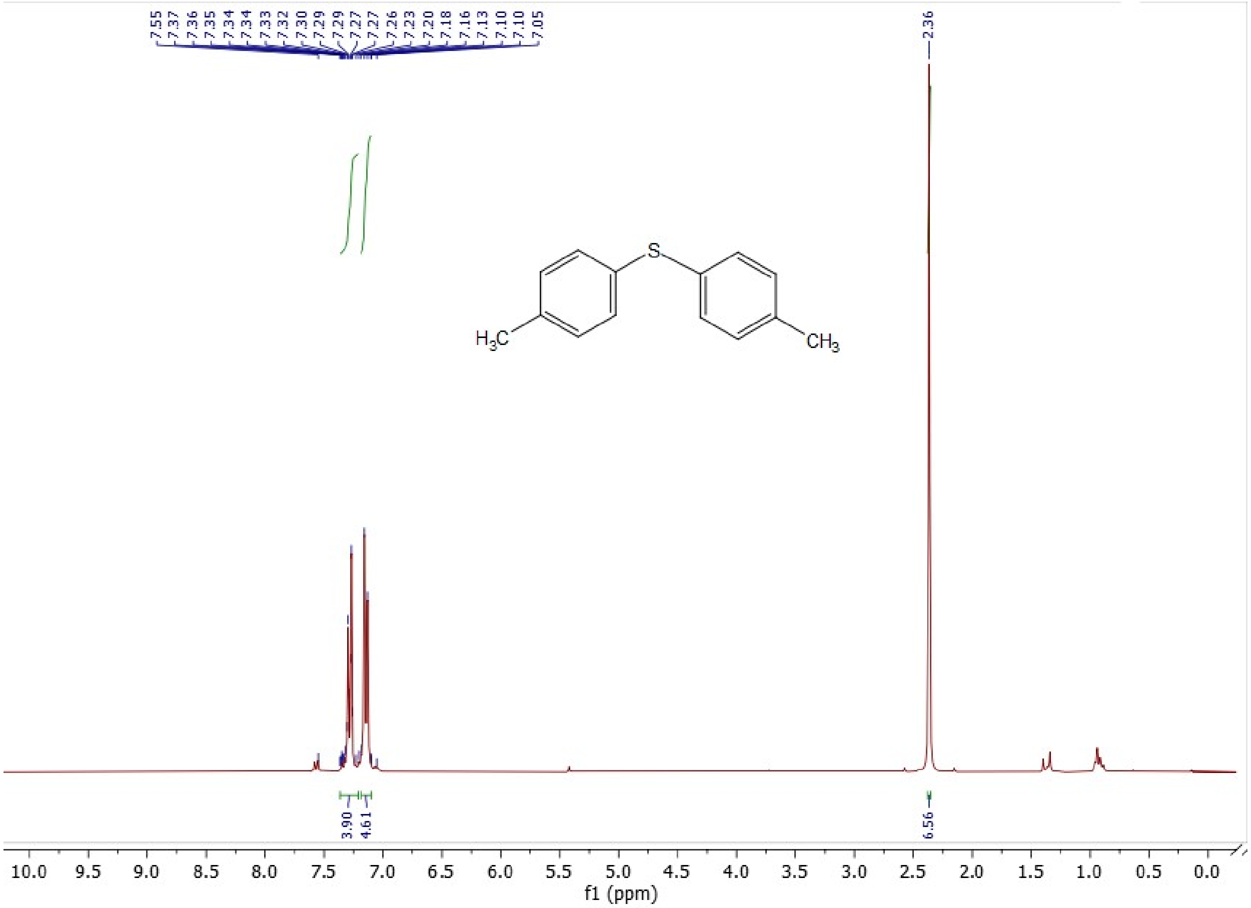

^1H NMR spectrum of Di(p-tolyl) sulfide, in $CDCl_3$

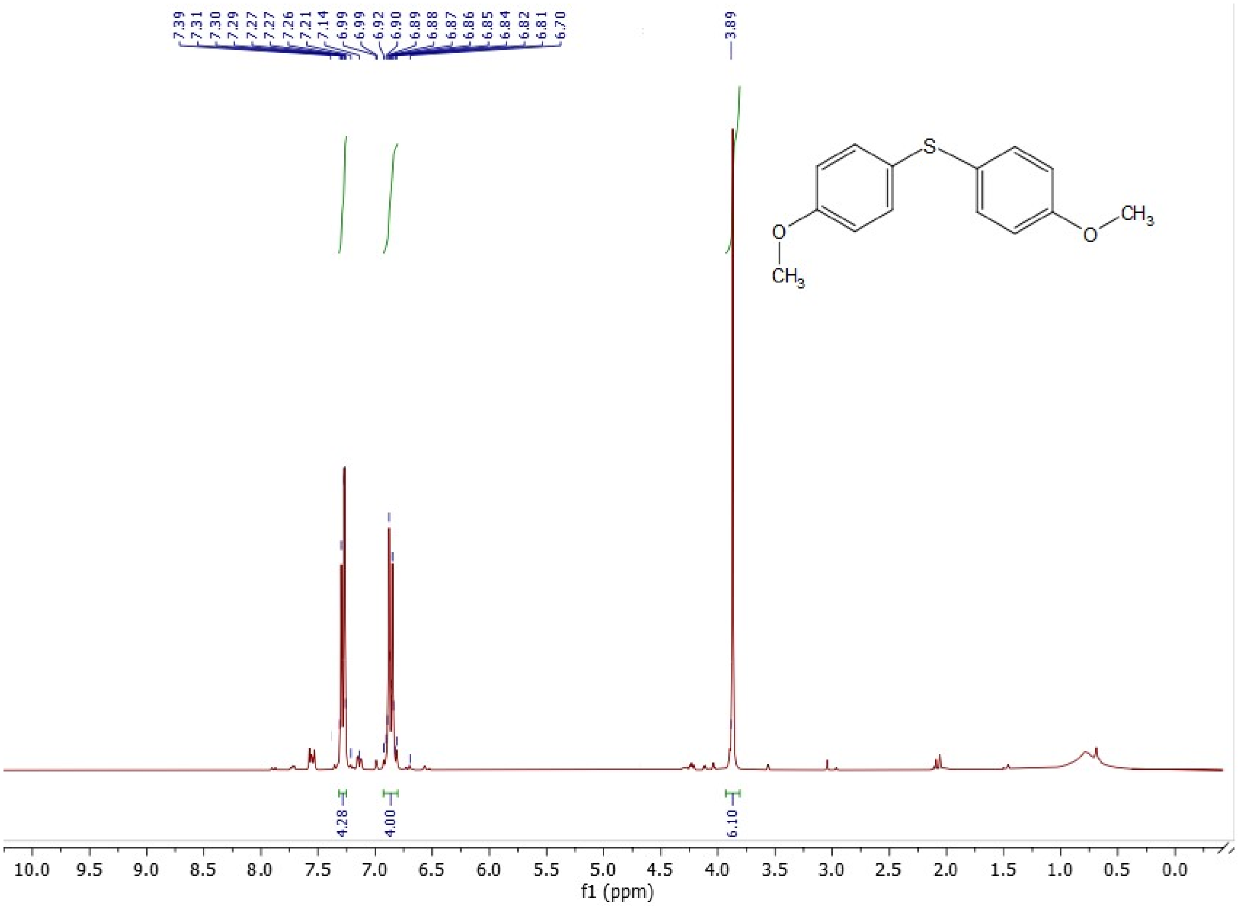

^{1}H NMR spectrum of 4,4'-Dimethoxy diphenyl sulfide, in $CDCl_3$

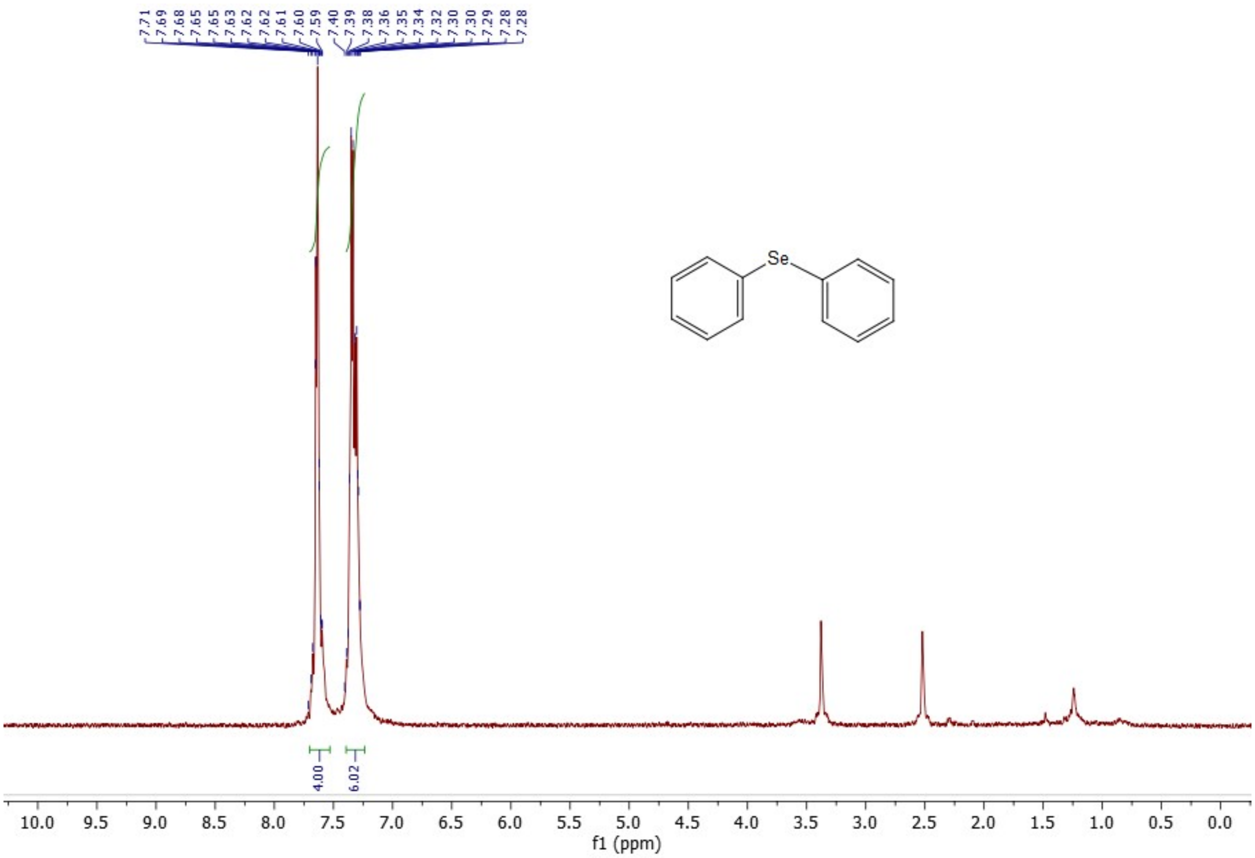

^{1}H NMR spectrum of Diphenyl selenide, in DMSO

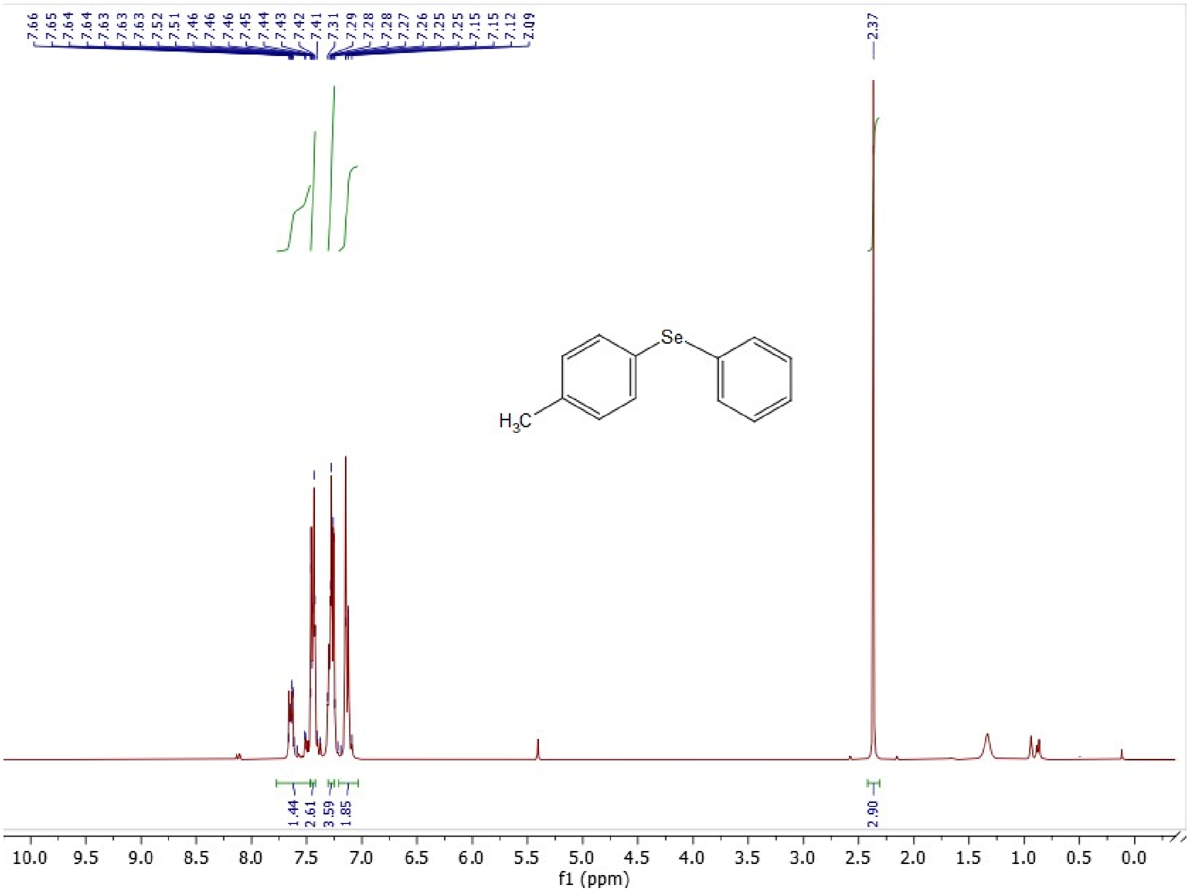

^{1}H NMR spectrum of Phenyl (p-tolyl) selenide, in $CDCl_3$
